# Expansive discovery of chemically diverse structured macrocyclic oligoamides

**DOI:** 10.1126/science.adk1687

**Published:** 2024-04-25

**Authors:** Patrick J. Salveson, Adam P. Moyer, Meerit Y. Said, Gizem Gӧkçe, Xinting Li, Alex Kang, Hannah Nguyen, Asim K. Bera, Paul M. Levine, Gaurav Bhardwaj, David Baker

**Affiliations:** 1. University of Washington, Department of Biochemistry, Institute for Protein Design, Seattle, WA 98195, USA; 2. University of Washington, Department of Medicinal Chemistry, Institute for Protein Design, Seattle, WA 98195, USA

## Abstract

Small macrocycles with four or fewer amino acids are among the most potent natural products known, but there is currently no way to systematically generate such compounds. We describe a computational method for identifying ordered macrocycles composed of alpha, beta, gamma and 17 other amino acid backbone chemistries, and use it to identify 14.9 million macrocycles with over 42,000 monomer combinations. We chemically synthesized 18 macrocycles predicted to adopt single low-energy states and determined their X-ray or NMR structures; 15 of these were very close to the design models. We illustrate the therapeutic potential of the newly discovered macrocycles by developing selective inhibitors of three protein targets of current interest. By opening up a vast space of readily synthesizable drug-like macrocycles, our results should considerably enhance structure based drug design.

Macrocycles composed of combinations of alpha, beta, gamma, delta, epsilon, aminobenzoic, aminophenylacetic, aminomethylbenzoic, and aminomethylpicolinic acids along with oxazoles, thiazoles, oxazolidines, thiazolidines, triazoles and thioethers have bioactivities ranging from antifungal or antibiotic properties to cancer cytotoxicity to pain relief (1–4). Despite the diversity of bioactivities observed in natural products of this class, exploration of this space has been largely limited to variants of those present in nature—indeed, the majority of macrocyclic drugs currently approved for use in humans are derived from natural products(5–7). The exploration of macrocycle chemical space in large-scale display techniques is largely constrained to those composed of primarily alpha amino acids (8, 9). While current diversity-oriented synthesis techniques in principle enable the combinatorial synthesis of 10s to 1000s of macrocycles from more exotic building blocks, these approaches are challenged by limitations in available chemistries (10–13).

Computational methods that enable the rapid and comprehensive exploration of the space of possible macrocycles could greatly facilitate the discovery of natural product-like compounds with novel bioactivities, but existing methods are similarly constrained by the diversity of chemistries accessible to them. Generative models derived from AlphaFold and RosettaFold enable designing small peptides composed of only the 20 canonical amino acids (14, 15). Enumerative methods that rely on random sampling of backbone torsion angles to identify closed conformations of macrocycles become intractable when attempting to enumerate macrocycles built from more than a single backbone building block (16, 17). There are nearly 60,000 two dimensional chemical structures of 2-, 3-, and 4-residue macrocycles that can be constructed from the 22 building blocks depicted in Figure 1A, and each of the 60,000 can be further diversified with potentially millions of combinations of different sidechains. This chemical space is far too large to rely on random sampling to identify the small fraction of linear sequences that can be closed to form a cycle.

We set out to develop a computationally tractable method for sampling the very large chemical space of possible small macrocycles (Figure 1; see Methods). We devised a three step approach for carrying out this search given an input set of monomers with widely varying chemical structures. In the first step, we use an adaptive grid based search over the AIMNet (18, 19)) potential energy landscape for each monomer to identify low energy conformers. In the second step, the rigid body transformations associated with these monomer conformations, and all dipeptide conformations constructed from pairwise combinations of these monomers, are computed and stored in hash tables. In the third step, macrocycles are rapidly and systematically generated by identifying pairs of entries in the hash tables for which the combination of the rigid body transforms is close to zero (so that together the fragments form a cycle). This transform based approach allows rapid identification of two, three or four residue macrocycles from every unique combination of the monomers depicted in Figures S1 in far less compute time than would be required to explicitly build backbone coordinates and evaluate closure for each of the different monomer combinations. The approach is agnostic to the number, identity, and connectivity of atoms between the terminal amides, as only their relative orientations in space are considered for determining closure.

## Enumeration of closed macrocycles

We used our approach to explore the very large space of 3- and 4-residue macrocycles constructed from 130 unique monomers, and their enantiomers where appropriate. These monomers fall into 22 classes, hereafter referred to as chemotypes, which are distinguished by the atomic number and hybridization of the atoms in the backbone of each monomer (Figure 1A); we refer to each chemotype by a letter (e.g. a for alpha-amino acids, b for beta-amino acids, d for delta-amino acids, etc; see Figure 1A legend). We constructed hash tables for all low energy conformers identified from the potential energy surfaces for each of the 130 monomers (Figure S1), and from dimers constructed from these low energy conformers, and then systematically searched through all combinations of monomer-dimer, dimer-monomer, and dimer-dimer hash tables, generating ensembles for each chemical for which matching hash values could be found. These hash tables contain nearly 9 billion unique conformers, and generating all combinations explicitly to check for closure would require building *ca.* 10^19^ macrocycles; our hashing approach reduces the complexity required to evaluate all of these combinations from O(n2) to O(n).

Systematically searching through the hash tables yielded 14.9 million closed macrocycles containing 9- to 32-membered rings belonging to 3,494 3-residue and 38,544 4-residue chemotypes (accounting for all unique circular permutations of the cyclic chemotypes). The chemical space spanned by the 3- and 4-residue chemotypes greatly expands upon what has been previously explored (Figure 2A and S3): 206 unique chemicals belonging to 23 chemotypes have high resolution structures deposited in the Cambridge Structural Database (CSD) and 13,932 chemicals belonging to 397 chemotypes are described in the pubchem database. Previous computationally designed macrocycles have been built from just one chemotype (alpha amino acids).

Each chemotype samples distinct three dimensional shapes (Figure 2B and C, S5). To characterize the structural diversity for each chemotype, we binned backbone torsion angles into 60 degree bins, and represented each macrocycle conformer by a string of these bins. The many thousands of conformers generated for the macrocycles belonging to each chemotype for the most part have only a handful of bin strings, which likely reflect the torsional preferences of the constituent amino acids. Principal moment of inertia (PMI) analysis of conformers spanning all the bin strings sampled for each sequence revealed that different macrocycle chemotypes sample distinct regions of shape-space (20). In some cases —e.g. aaaa and aaac— the specific 3D shapes sampled are quite restricted by the closure constraint, regardless of sequence or torsional diversity.

The hash-based closure generates for each monomer sequence an ensemble of macrocycle structure models built from low energy monomer conformers. Given sufficient compute power, for each of these ensembles, we could carry out minimization in AIMNet (to incorporate monomer-monomer interactions, closure strain, etc), and evaluate the degree to which the sequence of monomers uniquely encodes a single low-energy conformer by considering the energy landscape mapped by the set of all low energy conformers for the sequence (using for example the P_near_ approximation to the Boltzmann weight; (21)). Indeed, we found that the hash sampling procedure coupled with AIMNet calculations can predict the structures of small macrocycles from their sequence of monomers with backbone RMSDs between the predicted and experimental structures less than 0.3Å (Figure S6). Carrying out the full energy calculations on all 14 million generated ensembles is, however, not computationally tractable: approximately 10^8^ cpu-hours would be needed to minimize the *ca*. 23 billion conformers sampled across the 14 million ensembles. Instead, we focused on two subclasses of closed macrocycles we hypothesized would be particularly likely to have single unique ground states: first, those with local interactions strongly favoring one or a small number of closed states, and second, those containing non-local hydrogen bonds between backbone amides.

## Locally encoded macrocycles

To identify the first locally-encoded subclass, we generated conformational ensembles from hash tables containing only monomers within 1 kcal/mol of their lowest energy state; such macrocycles are torsionally optimized from the perspective of the composite building blocks. As the resulting macrocycle space is still very large, for computational tractability we explored the use of the number of different torsion bin strings sampled in these ensembles as an indicator of the extent to which the torsional biases together with the closure constraint are sufficient to specify a single low energy minimum. We found following AIMNet minimizations of a subset of the ensembles that in cases where five or fewer torsion bin strings were sampled there were generally deep energy minima in the AIMNet landscapes around a single state with Pnear > 0.9 (Figure S7). We identified *ca*. 380,000 3- and 4-residue sequences for which the hash generated ensembles contained more than 50 closed conformers that spanned five or fewer torsion bins, and carried out AIMNet minimization on *ca*. 4800; roughly 85% of these had single low energy structures with Pnear > 0.9.

We synthesized 13 such macrocycles and were able to determine structures of 10 using X-ray crystallography and NMR spectroscopy (Figure 3, S8). Six macrocycles grew crystals in vapor diffusion experiments of sufficient quality for structure determination using direct methods; seven macrocycles had NMR spectra in DMSO-d6 with sufficient dispersion to enable the unambiguous assignment of key ROEs in ROESY spectra—for these we generated 3D models by relaxing conformers generated from integrated ROE based distance constraints in unconstrained molecular dynamics (MD) simulations, and selecting the lowest-energy conformers (see Methods). Eight of the 11 experimentally determined structures superimpose on their respective design models with RMSDs of 0.8 Å or less, with small differences in ring puckering of 5- and 6-membered rings, in single side chain torsion angles, and in pairs of backbone torsion angles (larger differences were observed for the other three macrocycles (Figure S8)). We were able to determine both X-ray crystallographic structures and NMR structures for two of the 11 macrocycles. In these two cases both the NMR structure and the X-ray crystallographic structures are very similar to their respective design models and to each other (Figure S9).

The ten structures (Figure 3) are built from a total of eight different monomer chemotypes arranged into six unique macrocycle chemotypes. Design aas **1** contains two hydrogen bond interactions; one between backbone amides; the second involving the primary amide of the s monomer. Both aaam macrocycles and macrocycle akak **1** contain proline-like monomers sampling *cis* amides. In the NMR structures of both aaam macrocycles these motifs lead to strong ROEs between the C𝛼 proton of the proline-like monomer and the C𝛼 proton of the monomer immediately *N*-terminal to it; these macrocycles contain ɑ-amino acids in very extended β-sheet-like conformations and the *cis* amides rapidly turn the backbone to enable closure (Figure S10). Macrocycle aaaq **1** contains an extended pentafluorophenylalanine residue that buries its amide NH against the 4-aminomethylphenylacetic acid thereby shielding it from solvent and causing the amide NH to shift downfield with a temperature shift coefficient (Tcoeff) of 0.9 ppb/K (the backbone amides in the remaining monomers of this design shift upfield with Tcoeffs all less than −5.0 ppb/K, see supplementary data). Both aaam designs and aaaq **1** provide two different ways to mimic the extended conformations of poly-ɑ-amino acid peptides, like those adopted by many protease and kinase substrates (22, 23), and could be useful for targeting these enzymes.

## Hydrogen-bond stabilized macrocycles

To identify macrocycles containing transannular hydrogen bonds we constructed dimer hash-tables with conformers for which the terminal amides make a hydrogen bond, increasing the monomer energy cut-off for hash table inclusion to increase sampling of these rare interactions. To reduce local strain, following identification of macrocycle chemotypes that contained long range hydrogen bonding interactions, we chose monomer identities and sidechain rotamers with low energy given the backbone torsion angles. Following hash table-based ensemble enumeration and AIMNet minimization, we selected for experimental characterization designs with much lower energy than predicted by a simple monomer composition based model (see methods).

We prepared 17 macrocycles designed to contain long-range hydrogen bonding interactions and were able to determine the high resolution structures of seven (Figure 4). Four macrocycles grew crystals in vapor diffusion experiments of sufficient quality for structure determination using direct methods; three macrocycles displayed NMR spectra with sufficient dispersion to facilitate the unambiguous assignment of key ROEs in ROESY spectra. Eight of the seventeen macrocycles displayed 1D-NMR in DMSO-d6 indicative of a mixture of states; six of these collapse into a single major species in CDCl_3_. While only three macrocycles showed sufficient dispersion for unambiguous assignment, ten of the 17 had sufficient dispersion to enable measuring amide Tcoeffs which are consistent with the presence of the designed transannular hydrogen bonds in all ten cases (supplemental data). All seven of the experimentally determined structures superimpose on their respective design models with RMSDs less than 0.8 angstroms. Like the macrocycles described in Figure 3, differences between the designed model and the experimental structures of these hydrogen bond-containing macrocycles involve differences in ring-puckering and 180 degree rotation of backbone amides.

The macrocycles depicted in figure 4 are constructed from eight different monomer chemotypes arranged into six unique macrocyclic chemotypes, and are stabilized by a transannular hydrogen bond between backbone amides involving non ɑ-amino acids. Macrocycle aalm **1** contains a hydrogen-bonding fragment built from monomers with predominantly sp2 hybridized atoms in the backbone (lm), macrocycle aagb **1** contains a hydrogen-bonding fragment whose backbone contains many more sp3 hybridized atoms than present in ɑ-amino acid backbones (gb), and macrocycle aabi **2** contains a hydrogen-bonding fragment that blends these two features (bi). Six of the macrocycles contain contiguous fragments of ɑ-amino acids that mimic β-turns common in protein-protein interfaces (24–27): the designed macrocycles belonging to the aagb, aabi, and aaam chemotypes contain turns akin to type-I β-turns, and the aalm-based macrocycle contains a turn akin to the type-II β-turn. The *N-*methylated amino acid residues present in both aaap-based macrocycles adopt *cis* amides, resulting in type-VI like turns. Despite the presence of these obvious β-turn-like features in aaam **6** macrocycle, the spacing and orientation of the two phenylalanine side chains mimic the spacing and orientation of side chains positioned at i and i+4 of an ɑ-helix (Figure S11; this spacing is not present in either aaap **3** or aaap **4** which are isomers of aaam **6** at the level of the backbone atoms). This mimicry of beta turn and helical arrangements should be useful in targeting proteins recognizing these structural elements.

We measured the passive membrane diffusion of all 29 macrocycles designed with the two approaches using the parallel artificial membrane permeability assay (PAMPA) (Figures 3, 4, and S12; roughly half of the 14.9 million closed macrocycles satisfy the rule-of-five (Ro5) criteria for drug-like compounds, Figure S4 (28)). Almost all of the macrocycles were quite membrane permeable: 16 of the 29 had log(P_app_)’s greater than −6, and only three were not detected in the acceptor well. Under the same conditions, the small molecule drug propranolol achieved log(P_app_) of −5.36 (Figure S12). As expected, designs with exposed polar groups, particularly side chain hydroxyls and primary amides, had lower permeabilities. Overall, the hydrogen-bond containing designs were more permeable than the torsionally-optimized based designs, likely due to fewer exposed backbone polar groups(29–31). The vast majority of the macrocycles are stable to serum proteases; the half-lifes in reconstituted serum of all but a single macrocycle were greater than 24 hours (Figure S19).

## Designing macrocyclic inhibitors of protein targets

We sought to utilize the above methodology to identify chemically diverse macrocycles capable of inhibiting protein targets of interest starting from known inhibitors with limited potency and/or selectivity. We used a motif scaffolding approach to identity inhibitors of histone deacetylase 6 (HDAC6) and the main protease of SARS-CoV-2 (Mpro). We started from dipeptide fragments derived from the previously described crystal structure of des4.3.1 bound to HDAC6 and the crystal structure of the linear *C*-terminal prosequence bound to Mpro (see methods) (32, 33). We identified candidate macrocyclic inhibitors by rapidly searching for closures across all hydrogen-bond containing hash tables with these dipeptides, building the resulting 4-residue macrocycles in their respective pockets, and discarding candidates that clash with the surrounding protein residues. This search embeds the starting dipeptide motif in nearly 38,000 possible macrocycles whose 3D coordinates are constructed in the context of the target protein binding site. We prioritized synthesizing candidate macrocycles based on three criteria: the degree to which the designed sequence preorganizes the dipeptide fragment into the binding-competent conformation assessed by Pnear analysis of the generated ensembles, recovery of the designed complex in ligand docking simulations performed in GALigandDock (34), and predicted binding free energy of the designed sequence compared to that of initial dipeptide motif. After filtering the candidate macrocycles with the above criteria, we synthesized 11 macrocycles belonging to 6 chemotypes designed to inhibit HDAC6 and 6 macrocycles belonging to 5 chemotypes designed to inhibit Mpro.

Ten of the eleven chemically synthesized macrocycles displayed IC50s against HDAC6 ranging from 1.5 nM to 70 nM, a 10-fold to 400-fold improvement in potency compared to the minimal alkylthiol warhead present in each design (Figure 5A-C, S20, Table S11). The most selective of these macrocycles belong to three distinct chemotypes and are 100-fold to >1000-fold more selective for HDAC6 over the other HDACs tested. The observed selectivity of the designed macrocycle is comparable to several advanced HDAC6-selective inhibitors (SI Table 11). Of the 6 designed Mpro inhibitors, only one belonging to the aabi chemotype displayed a modest IC50 of *ca.* 16 μM (Figure 5D, S21, SI Table12). We subsequently constructed and redocked a focused *in silico* library of homologues of this modest inhibitor using GALigandDock keeping the backbone chemistry and its predicted structure unchanged and varying the sidechains. From this *in silico* library we selected and chemically synthesized 27 homologues; 17 of which displayed IC50s against Mpro ranging from 4 μM to 0.88 μM (SI Table 12). Linear analogues of three active designs showed IC50s greater than 200 μM supporting the importance of macrocyclization of the sequence.

We next sought to identify macrocycles that inhibit the interaction of Bak and MCL1 using the large number of hash-generated macrocycle ensembles as an *in silico* virtual library. We generated MCL1-directed virtual libraries by first modifying each macrocycle to contain a sidechain dichloro-benzene moiety (a functional group previously described to interact with MCL1 at the MCL:Bak interface (35)), then mutated the remaining positions of the macrocycles to a host of other ncAAs, peptoids, and β3-amino acids beyond those for which we explicitly generated energy landscape (see methods). From a virtual library of ca. 2.7 million compounds, we prioritized macrocycles for synthesis based on the calculated binding free energy resulting from GALigandDock and visual inspection. 3 of the initial 18 synthesized macrocycles showed inhibitory activity near or below 50 μM in the FRET assay (Table S13). We subsequently generated and docked a larger virtual library of *ca*. 27 million macrocycles and identified 12 active designs from the 30 synthesized macrocycles (Figure 5E, S22, S23, Table S13). We suspect that the higher *in silico* and experimental success of the larger library is due to a greater diversity of shapes represented (36); the small library contained macrocycles belonging to only 22 chemotypes, whereas the larger library contained macrocycles derived from 3,991 different chemotypes. The identified hits spanned 10s of μM to 100s of nM in IC50, with the most potent being 220 nM.

## Conclusion

Small macrocycles found in nature generally populate a mixture of conformers in solution. Our approach enables the rapid exploration of the large and chemically diverse macrocycle space to identify those that populate primarily a single conformer. The two strategies employed here —using conformationally restricted building blocks to favor specific geometries, or incorporating longer range hydrogen bonding interactions— both resulted in macrocycles with well defined structures (another strategy is to introduce large barriers to backbone bond rotation (37)). Our observation that the chemotype of a macrocycle, regardless of its side-chain identity, largely defines its globular shape suggests that diversity oriented synthesis methodology seeking to span a wide range of shapes should focus on expanding the number of chemotypes that can be synthesized combinatorially as opposed to the diversity of side chains arrayed on a single or small number of chemotypes (the importance of macrocycle chemotype diversity has been noted in DNA-encoded libraries (38) ). Encouragingly for future therapeutic applications, nearly half of the macrocycles we characterized are membrane permeable, with log(Papp) > −6.0 in PAMPA, and this fraction would likely be considerably higher if we had explicitly designed for permeability, for example by disfavoring compounds with exposed NH groups. The chemical space can readily be expanded by including more diverse sidechains or additional non-canonical backbones in the hashing step, and almost all compounds in this class can be readily synthesized using standard manual Fmoc-based solid-phase peptide synthesis followed by solution-phase cyclization (see SI methods). Our binder design results show that diverse biochemical functions can be introduced into *de novo* designed small macrocycles with binding selectivies (as in the HDAC6 case) rivaling those of advanced small molecule therapeutic candidates.

The very large and diverse set of drug-like compounds described here opens up exciting new avenues in drug discovery. The rigidity of the molecules could translate into higher on-target affinity due to the lower entropy loss upon target binding, and lower off-target binding as there are fewer alternative states(39–43); rule-of-5 compliance during generation should lead to membrane permeability and other desirable pharmacological properties. Libraries of rule-of-5 compliant macrocycles populating single states can be screened in silico and/or experimentally to identify new lead compounds binding targets of interest. For targets for which small molecule fragments are already known to bind, custom rigid macrocycle libraries incorporating this specific functionality can be readily generated by searching the hash tables for all closed macrocycles that incorporate the fragments. These approaches should allow systematic discovery of new macrocyclic therapeutic candidates.

## Supplementary Material

Supplementary Material

**Supplementary Content:** Materials and Methods, Figs. S1 to S23, Tables S1 to S14, analytical characterization data, NMR spectral data, and references 45 – 52.

## Figures and Tables

**Figure 1.**
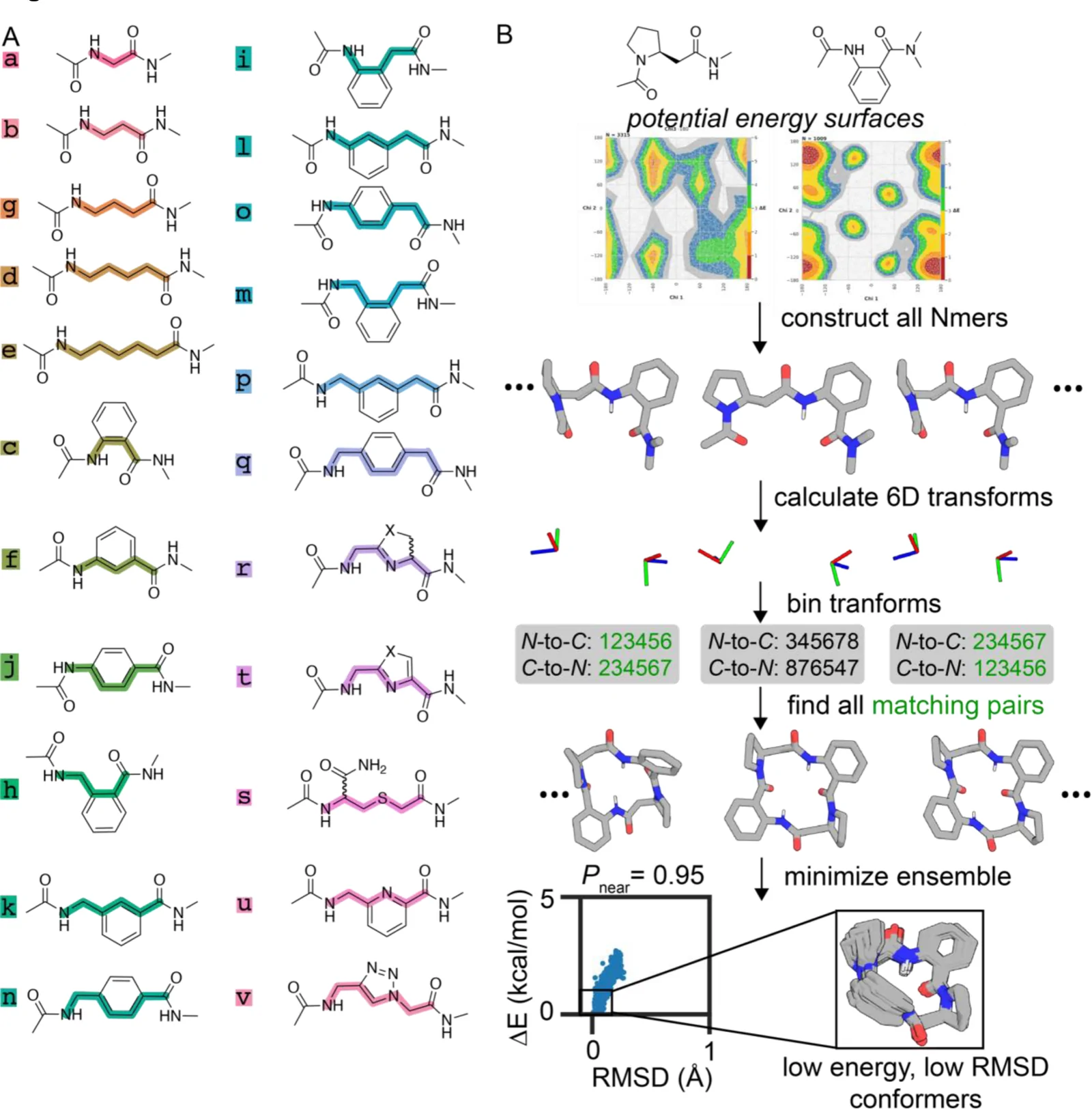
Overview of macrocycle discovery approach. (**A**) Chemical structures of monomeric chemotypes used to construct small drug-like macrocycles: amino acids (a, b, g, d, e), aminobenzoic acids (c, f, j), aminomethylbenzoic acids (h, k, n), aminophenylacetic acids (i, l, o), aminomethylphenylacetic acids (m, p, q), oxazolidines/thiazolidines (r), oxazole/thiazoles (t), thioethers (s), aminomethylpicolinic acids (u), and triazoles (v). The backbone atoms are colored to highlight the differences between chemotypes. The elaborated monomers used throughout this work that belong to these chemotypes are presented in Fig. S1. (**B**) Assembly and identification of low-energy conformers of cyclo-(βpro-ben2-βpro-ben2) beginning from calculated potential energy surfaces of Ac-βpro-NHMe and Ac-ben2-NMe2. Combining these low-energy conformers yields thousands of Ac-βpro-ben2-NMe2 dimers. The 6D transform between the planes defined by the terminal amides is calculated for each dimer, and then binned. All pairs of dimers that close one another (indicated by green in this example) are identified in a quick lookup and the resulting ensemble of macrocycle conformers is minimized.

**Figure 2.**
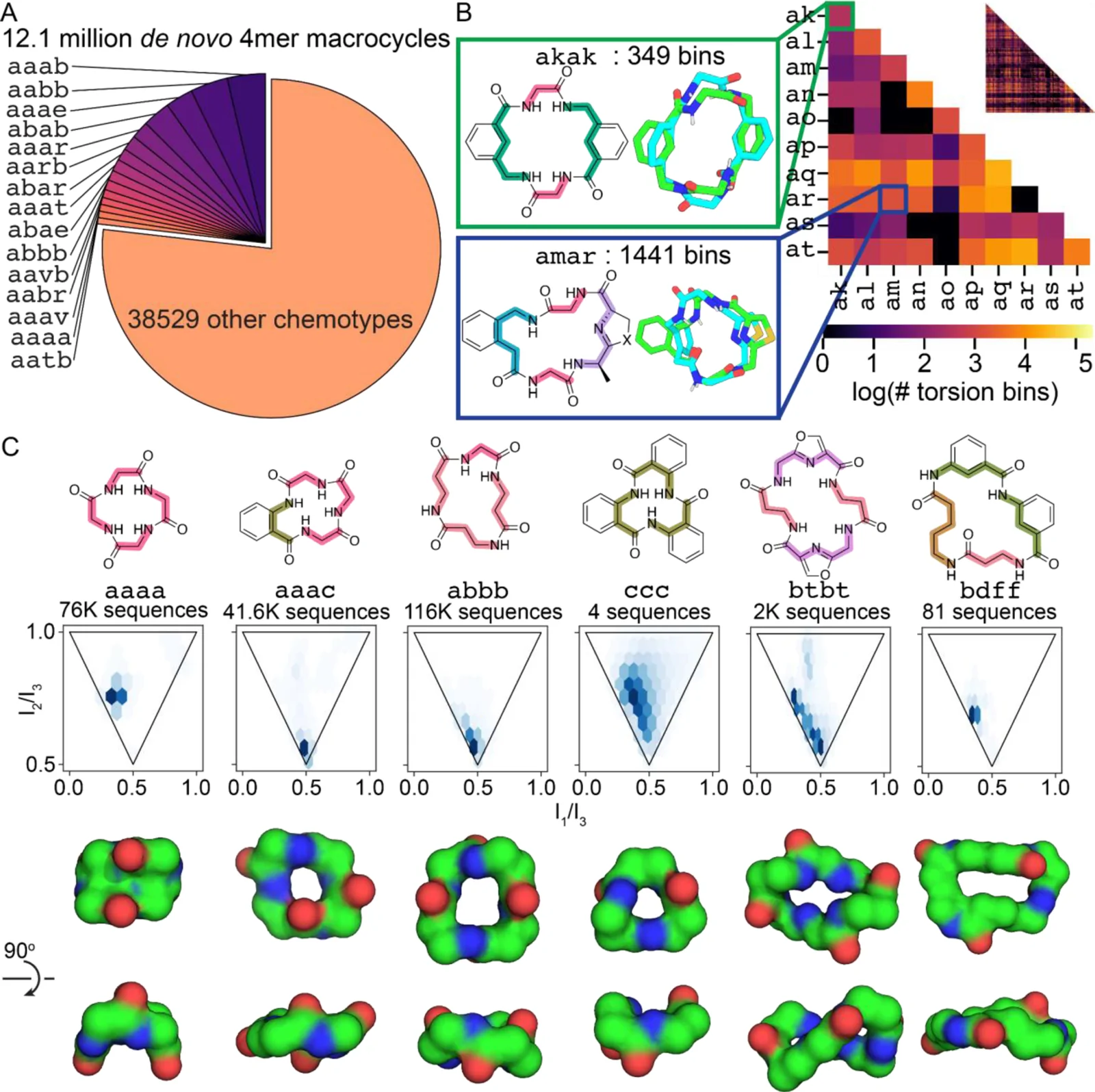
Chemical and structural diversity of small *de novo* macrocycles. (**A**) Distribution of unique sequences sampled across all 4-residue macrocyclic chemotypes. The 15 most populated chemotypes are labeled. (**B**) Torsional diversity of sampled 4-residue macrocycle chemotypes. Heat map pixels represent 4-residue macrocycle chemotypes generated from the two residue fragments on the x and y axes, and are colored by the number of unique backbone torsion bins sampled across all sequences belonging to that chemotype. The inset at top right shows the full map over all 22^4^ monomer chemotype combinations; a 10 × 10 subset of this is blown up in the main panel for clarity. Examples of two representative torsion bins are shown in the boxes at left for two unique chemotypes. (**C**) Principal moment of inertia distributions (second row) and representative conformers (bottom) sampled by macrocycles constructed from different chemotypes (top row).

**Figure 3.**
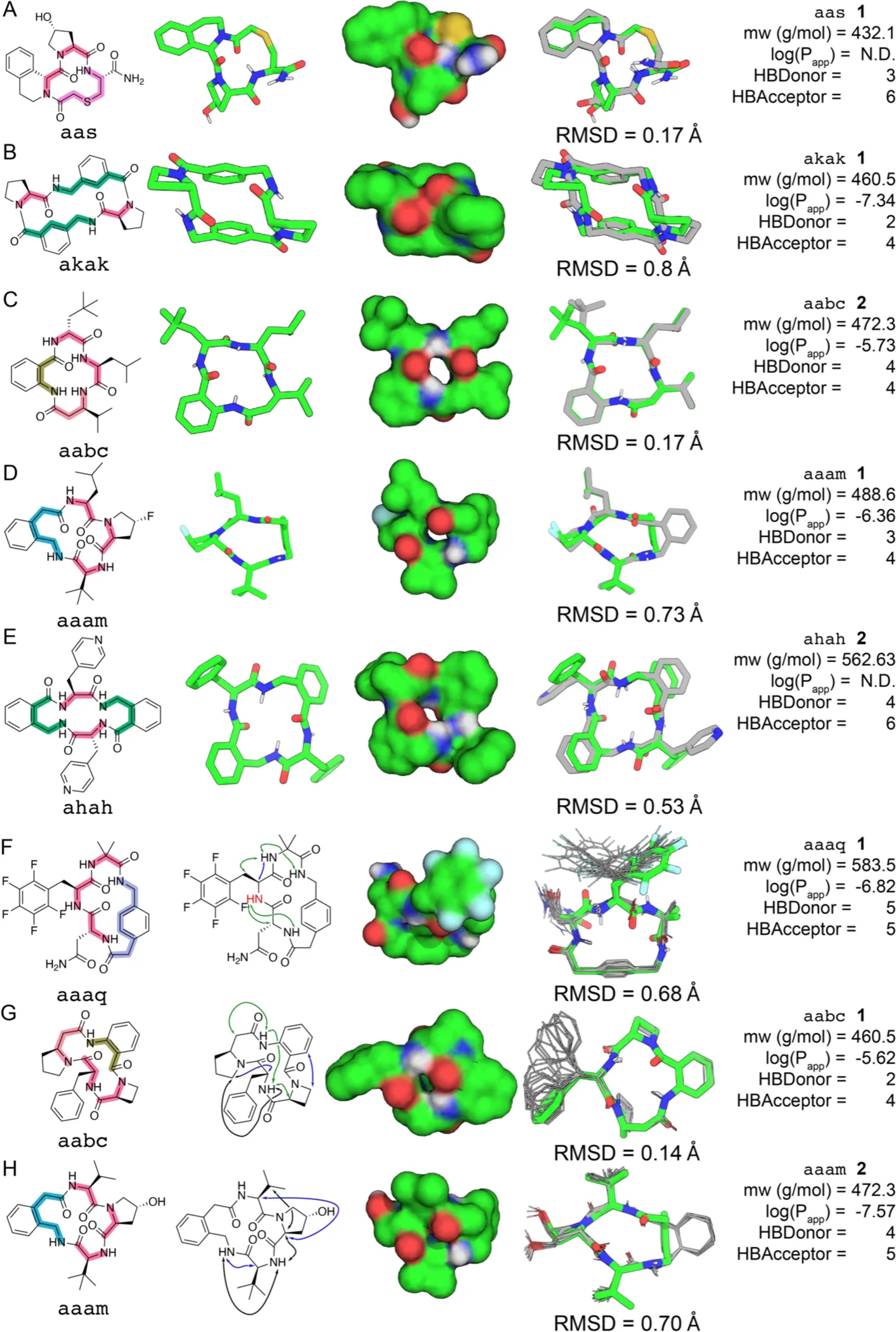
X-ray and NMR structures of locally encoded macrocycles are very close to the design models. Rows A-E crystal structures. Column I, chemical structure colored by chemotype; Column II design model; Column III: Surface representation of design models; Column IV: Superposition of the design model (green) and experimentally determined coordinates (grey); Column V: chemical properties and apparent permeabilities; the number of hydrogen bond donors (HBDonor) and hydrogen bond acceptors (HBAcceptor) was determined using rdkit. ahah **2** was modeled with phenylalanines and chemically synthesized with pyridylalanines (row E). Rows F-H, NMR structures. In column II, in place of the design model we show ROEs used for distance-constrained ensemble generation. Blue arrows, distances less than 2.5 A; green arrows, distances between 2.5 and 3.5 A; black arrows distances greater than 3.5 A. Experimental structure to design model RMSDs are reported over all backbone atoms for X-ray structures, and over ensemble average backbone coordinates for NMR structures.

**Figure 4.**
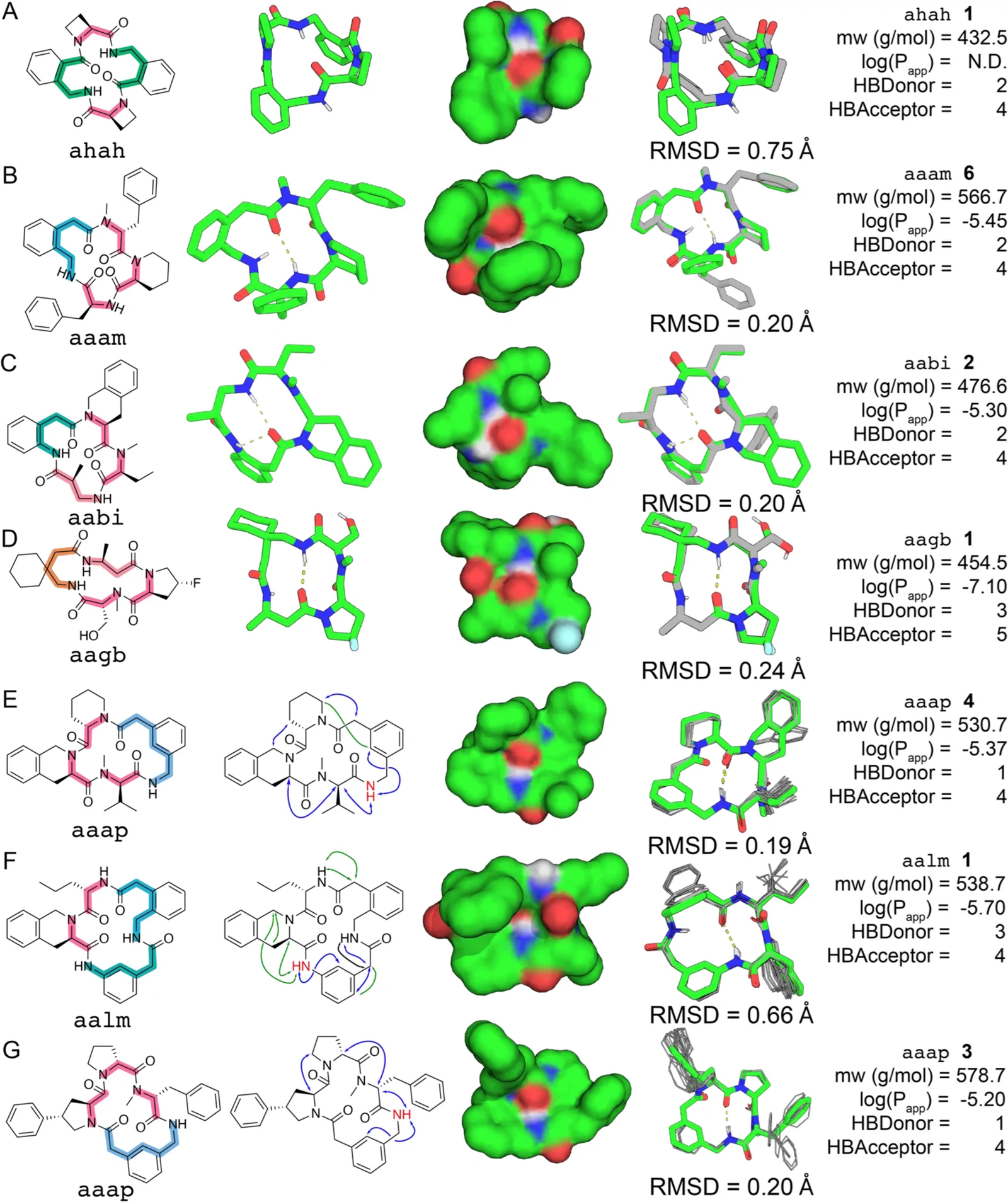
X-ray crystallographic and NMR ensemble structures of hydrogen bond-containing macrocycles are very close to their design models. Columns I-V are as in figure 3. Column II rows E-G: ROEs used for distance-constrained ensemble generation. Arrows are as in figure 3. Red amide NH indicates protons with Tcoeffs > −4.5 pbb/K.

**Figure 5.**
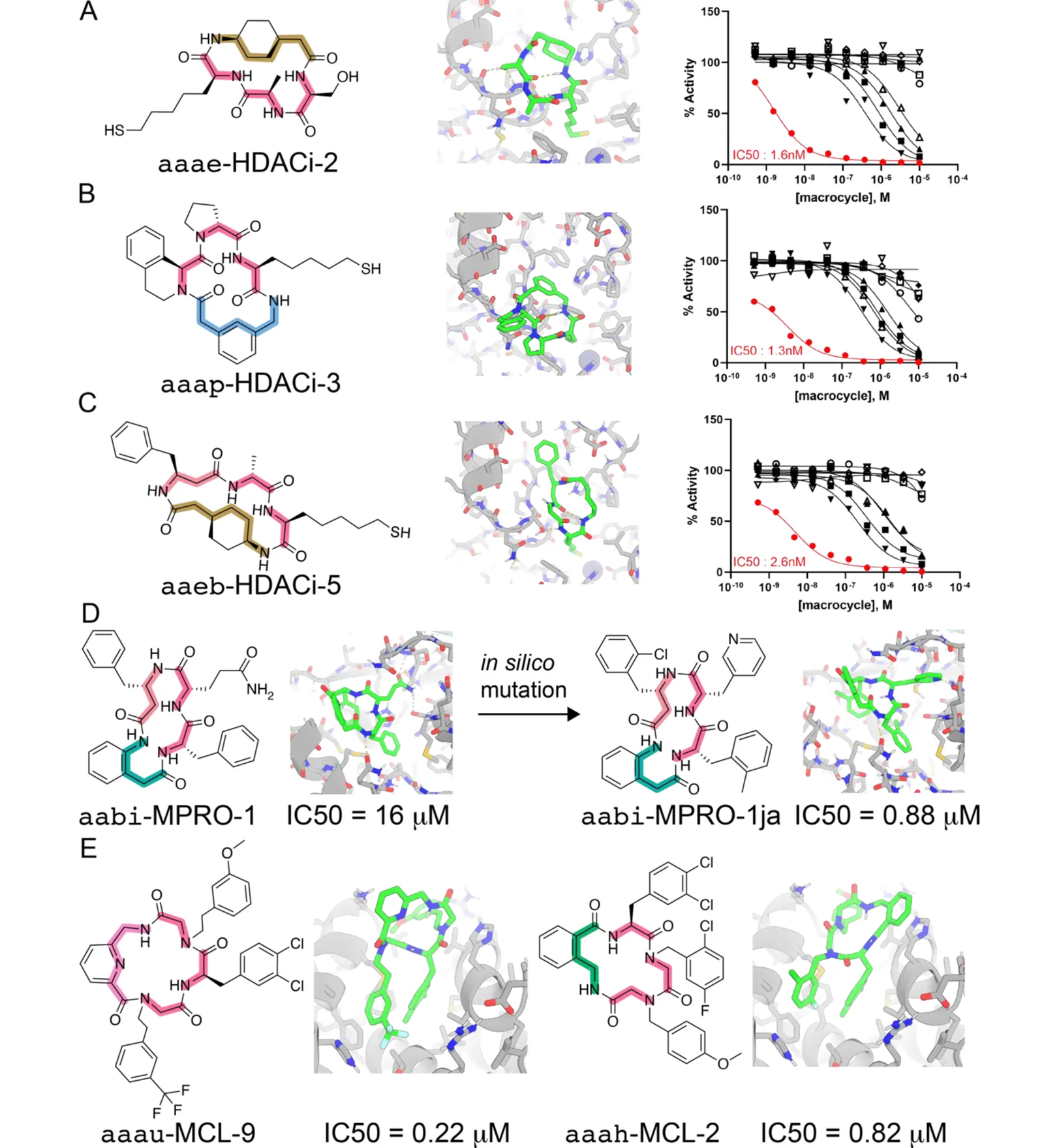
*De Novo* design of macrocycles that inhibit enzymes and protein-protein interactions. (**A-C**) Chemical structures (left), design models (middle) and concentration dependence of inhibition (right) of HDAC6 (red), HDACs 1 (closed square), 2 (closed triangle), 3 (closed inverted triangle), 4 (closed diamond), 5 (open circle), 7 (open square), 8 (open triangle), 9 (open inverted triangle), 11(open diamond) by designed macrocyclic HDAC inhibitors (HDACi). The macrocycles were designed to contain a nonselective alkylthiol moiety. The designed macrocycles show selectivity for HDAC6 over other HDACs ranging from 100-fold to greater than 1000-fold. (**D**) Chemical structures and design models of the initial hit vs Mpro (left) and most potent (right) designed macrocycle identified from a focused *in silico* library of mutants of the initial hit. (**E**) Chemical structures and design models of two of the most potent designed MCL1 : Bak inhibitors PPI inhibitors.

## Data Availability

All data are available in the main text or as supplementary materials. X-ray crystallographic structures were deposited into the Cambridge Crystallographic Data Center, under deposition numbers CCDC 2130362 (aas 1), 2130361 (akak 1), 2130363 (ahah 1), 2130360 (aabc 2), 213059 (aaam 1), 2130358 (aaam 6), 2130356 (aabi 2), 2130354 (aagb 1), 2130352 (bbbb 3), and 2203552 (ahah 2). All design scripts are available in supplementary file S1 and through Zenodo (44).
