# Supplementary material for: Expansive discovery of chemically diverse structured macrocyclic oligoamides
Source: Science. Author manuscript; Available in PMC 2026 Nov 13. (PMC13763402; doi:10.1126/science.adk1687)
Supplement: Supplementary file 1 [file NIHMS2211220-supplement-Supplementary_Material.docx]

Supplementary Materials for

Expansive discovery of chemically diverse structured macrocyclic oligoamides

Patrick J. Salveson, Adam P. Moyer, Meerit Y. Said, Gizem Gӧkçe, Xinting Li, Alex Kang, Hannah Nguyen, Asim K. Bera, Paul M. Levine, Gaurav Bhardwaj, David Baker

Correspondence to: [dabaker@uw.edu](mailto:dabaker@uw.edu)

**This PDF file includes:**

Materials and Methods

Figs. S1 to S24

Tables S1 to S15

Analytical HPLC and LCMS mass spectra of macrocycles

Variable temperature ^1^H NMR studies of macrocycles

^1^H NMR studies of macrocycles

References

**Other Supplementary Materials for this manuscript include the following:**

File S1 Scripts

Materials and Methods

Experimental Methods

*Peptide synthesis*: The macrocycles described here were prepared by WuXi apptec. Typically, WuXi assembled peptides on CTC resin using Fmoc-based solid-phase synthesis using iterative coupling with either HBTU/DIEA, HATU/DIEA, or DIC/HOBt. Linear peptides were cleaved from resin and then cyclized in solution. Typically, peptides were cyclized in either DCM or DMF using either TBTU/DIEA, HATU/DIEA or EDCl/HOBt. The crude cyclization reactions were then purified using reverse-phase HPLC. In WuXi’s hands, these procedures typically afforded 0.5 - 20 mg of purified macrocycle as white or off white powders, after lyophilization. We confirmed via LCMS that the isotope patterns and the measured z of observed ions were consistent with the desired macrocycle. The purity of each compound received from WuXi is summarized in Table S15. We have provided HPLC and mass spectra for each compound showing the isotope patterns of relevant ions in following sections of the SI. We observe both M and 2M singly charged ions for 39 of the reported compounds. The 2M ions may be the result of intermolecular cyclization. The remainder contain just singly charged M ions. Other impurities may have arisen from epimerization of the *C*-terminal residue in the cyclization reaction. Below is a more detailed protocol for the synthesis of aaap 4:

CTC resin (0.1 g, 0.1 mmol, 0.99 mmol/g) was swelled in DCM under agitation with nitrogen. A solution of Fmoc-3-aminomethyl-phenylaetic acid (38.74 mg, 0.1 mmol, 1.0 eq) in DCM was added to the resin and agitated. DIEA (4.0 eq) was added to the resin dropwise. The resulting mixture was allowed to mix under agitation for 2 hours. The solution was then drained, and the resin was capped with a methanol (0.1 mL) for 30 minutes. The loaded and capped resin was then washed in DMF. The linear peptide was assembled using iterative Fmoc-deprotection reactions using 20% piperidine in DMF followed by coupling reactions using 6 eq of the incoming Fmoc-protected amino acids, 5.7 eq of HATU, and 12 eq of DIEA. The resin was washed 5 times between each deprotection and each coupling reaction using DMF. The progress of these reactions were monitored using the ninhydrin test. After the final Fmoc-deprotection reaction, the linear peptide was cleaved from resin using a 1% (v/v) solution of trifluoroacetic acid in DCM. The resin was subjected to two bouts of cleavage, each lasting 3 minutes. The combined filtrate containing the cleaved linear peptide was then diluted into 100 mL of DCM to which HATU (76 mg, 2 eq) was added. The pH of this solution was adjusted to pH ~ 8.0 using DIEA. The resulting mixture was stirred at room temperature for 0.5 h after which 1 M HCl was added. The organic phase was collected and concentrated under vacuum to give the crude macrocycle aaap 4. The crude reaction mixture was dissolved in water:ACN and purified by reverse-phase preparative HPLC (25-55% ACN over 55 minutes. Pure fractions were combined and lyophilized yielding 14.1 mg of aaap 4 at ca. 99.4% purity.

*NMR Spectroscopy*: 1D ^1^H-NMR and 2D ^1^H-NMR spectra were recorded on a Bruker NOE 600 MHz spectrometer equipped with a QCI-F cryoprobe. TOCSY spectra were recorded with a 80-ms spin-lock mixing time. ROESY spectra were recorded with a 200-ms spin-lock mixing time. Data were processed in TopSpin.

*VT-NMR*: Variable temperature NMR experiments were performed to determine amide hydrogen temperature shift coefficients. 1H-NMR spectra were recorded from 298 to 318 K in either DMSO-d6 or CDCl3. Temperature shift coefficients less than 4 ppb/K were inferred to represent hydrogens that are shield from solvent, whereas coefficients greater than 4 ppb/K were inferred to represent hydrogens exposed to solvent.

*NMR-based structure determination*: The 3D structures of peptide macrocycles were determined using NMR-based distance constraints in combination with molecular dynamics simulations. ROE-based distance constraints were determined from the averaged integrated columns of ROE cross peaks across the diagonal. These integrals were converted to atomic distances using an inverse 6th power relationship. A reference distance of 1.78 angstroms for diastereotopic methylene protons or 2.49 angstroms for 1,2-substituted phenyl protons was used to calibrate the conversion of integrated ROEs to atomic distances. The calculated distances were then adjusted by 10% to provide a range of allowed distances for each distance constraint. Ensembles of macrocycle conformers that obeyed each distance constraint were obtained using the geometric hashing scheme detailed above. First, we constructed hash tables that contained monomers up to 6 kcal/mol in energy above the global minima. We then searched for closures in these tables. This search produced 106-108 conformers of the sequences in question. We then filtered those conformers based on their satisfaction of all of the experimentally derived distance constraints, resulting in an ensemble of approximately 400 conformers of the peptide. Each of these 400 conformers was subjected to a 50 picosecond unconstrained molecular dynamics simulation using xTB at 298K in either implicit DMSO or implicit CHCl_3_ (45). In total this resulted in approximately 20 nanoseconds of simulation time for each peptide. The 20 lowest energy structures across the entire ca. 20 ns simulation were taken as the ensemble structure of the peptide.

*Single crystal X-ray diffraction of peptides*: Crystals diffraction data were collected from a single crystal at synchrotron (on APS 24ID-C) and at 100 K. Unit cell refinement, and data reduction were performed using XDS and CCP4 suites (46, 47). The structure was identified by direct methods and refined by full-matrix least-squares on F2 with anisotropic displacement parameters for the non-H atoms using SHELXL-2018/3 (48, 49). Structure analysis was aided by using Coot/Shelxle (50, 51). The hydrogen atoms on heavy atoms were calculated in ideal positions with isotropic displacement parameters set to 1.2 × Ueq of the attached atoms. Crystallographic structures were deposited into the Cambridge Crystallographic Data Center, under deposition numbers CCDC 2130362 (aas 1), 2130361 (akak 1), 2130363 (ahah 1), 2130360 (aabc 2), 213059 (aaam 1), 2130358 (aaam 6), 2130356 (aabi 2), 2130354 (aagb 1), 2130352 (bbbb 3), and 2203552 (ahah 2).

*PAMPA*: Stock solutions of each peptide were prepared gravimetrically by dissolving ca. 1 - 3 mg of peptide in either 100% MeOH or 100% DMSO to produce ca. 1-10 mM peptide solutions. These stock solutions were stored at 4 oC when not in use. Samples for PAMPA experiments and calibration curves were prepared by diluting these stock solutions to produce 1.2 mL of 20 uM peptide in PBS supplemented with either 5% DMSO, or 10% MeOH. Calibration curves of each peptide were prepared by serial diluting 20 uM peptide 2-fold to obtain 6 samples with the following concentrations: 20, 10, 5, 2.5, 1.25, and 0.625 uM. These standard curves were used to determine the concentration of each peptide in both the donor and acceptor wells of the PAMPA plate. For all PAMPA experiments, we used the commercially available Corning Gentest Precoated PAMPA plate system (product number 353015), following the manufacturer's instructions. Briefly, donor wells were loaded with 300 uL of the 1.2 mL solutions; acceptor wells were loaded with 200 uL of either 5% DMSO in PBS or 10% MeOH in PBS. Each peptide was prepared in triplicate. The concentration of each peptide in the donor and acceptor wells was quantified by LCMS after approximately 19 hours. These concentrations were used to calculate Papp per the manufacturer's instructions. Measurements were made on triplicate technical repeats on a single plate.

*Serum stability*: Stock solutions of the peptides (10 mM in DMSO) and reconstituted normal human serum (Thermo Scientific) in PBS were pre-equilibrated to 37 °C for 15 minutes in separate tubes. For each replicate, peptides and serum were combined to a final concentration of 50 μM and 50% (v/v) respectively, final volume of 60 μL. The reaction tubes were then incubated at 37°C in a Bio-Rad thermocycler with the lid temperature at 50°C to avoid condensation. At each time point, 10 μL of the reaction was taken from each replicate and diluted with three-fold excess of cold acetone. The serum proteins were allowed to precipitate for 15 minutes at -20 °C and were removed by centrifugation at 14,000 g, 4 °C for 10 minutes. 10 μL of the supernatant was transferred to a new tube, diluted with the same volume of water, and were stored at -20 °C until LCMS analysis. Chromatographic separations were performed using an Agilent LC-TOF system using a C18 reversed phase analytical column (Higgins Analytical, 150x4.6 mm, 5 μm pore size) using a 30-95% B linear gradient over 30 minutes. Solvent A consisted of 0.1% (v/v) formic acid in water, and solvent B contained acetonitrile, 0.1% formic acid. Peak areas corresponding to the intact peptide were obtained by manual integration using Agilent MassHunter acquisition software.

*Histone Deacetylase inhibition assays*: Assays were performed as a fee-for-service through ReactionBiology, from 10 mM DMSO stock solutions of each compound, using their standard conditions. Compounds were tested in a 10-dose IC50 singlet experiment with a 3-fold serial dilution starting at 10 uM. The two positive control compounds, trichostatin A and TMP 269, were similarly tested except starting at 10uM or 1 uM respectively. For HDACs1,2,3,6 the fluorogenic substrate was derived from p53 residues 3790382 (RHKK(Ac)AMC); for HDACs4,5,7,9,11 the fluorogenic substrate was derived from HAC class2a trifluoroacetyl lysine. For HDAC8, the fluorogenic substrate was derived from p53 residues 379-382 (RHK(Ac)K(Ac)AMC). Curve fits were performed when the activities at the highest concentration of compounds were less than 65%.

*Sars-CoV-2 Main protease inhibition assays*: Assays were performed as a fee-for-service through ReactionBiology, from 10 mM DMSO stock solutions of each compound, using their standard conditions. Briefly, compounds were tested in a 10-dose IC50 singlet experiment with a 3-fold serial dilution starting at 200 uM. The control inhibitor, GC376 was tested in a similar 10-dose experiment, starting at 1 uM. The protease activities were monitored as a time-course measurement of the increase in fluorescence signal from fluorescently-labeled peptide substrate, and initial linear portion of slope (single/min) was analyzed. Curve fits were performed when the activities at the highest concentration of compounds were less than 65%.

*MCL1 binding assay*: Assays were performed as a fee-for-service through Reaction biology , from 10 mM DMSO stock solutions of each compound, using their HTRF assay. The assay is based on the competition of fluorescently labeled peptide binding to MCL. The binding is monitored by HTRFsignals between anti-GST-Tb and FAM-Bak-peptide using GST-tag MCL. The competition assay was performed with 4 nM MCL1, 100 nM FAM-Bak-peptide in 20 mM potassium phosphate, 50 mM NaCl, 1mM EDTA, 0.005% triton X-100, 1.0 % DMSO at pH 7.5. Each compound was tested in a 10-dose IC50 singlet with 3-fold serial dilution starting at 50 uM. The positive control compound, A-1210477 was tested in a similar scheme, except starting from 1 uM. Compounds were introduced to MCL and allowed to incubate for 10 minutes, followed by the addition of FAM-Bak-peptide. After 10 additional minutes of incubation, anti-GST-tb was added and the HTRF signal was measured after 60 minutes. Curve fits were performed when the activities at the highest concentration of compounds were less than 65%.

Computational methods

The entire computational pipeline is implemented using several python packages. The following sections detail their execution and high-level details of their operations. The scripts themselves are available in supplementary file S1 or at Zenodo (*44*).

*Residue parameters*: To facilitate constructing and manipulating coordinates of both monomers and macrocycles, we generated a python dictionary that contains myriad parameters for each residue. These parameters store several boolean values concerning the chemical identity of the residue, python lists of various atom indices, and SMILES strings of the residue. These residue parameters are used in myriad places throughout the scripts and workflows below. The following is a simplified version of the residue_params dictionary that contains only glycine.

```
residue_params = {
    'AGLY' : {
        'smiles' : 'CC(=O)NCC(=O)NC',
        'dofs' : [[1, 3, 4, 5], [3, 4, 5, 7]],
        'proline_like' : False,
        'preproline' : False,
        'chiral' : False,
        'lower_smiles' : 'CC(=O)',
        'residue_smiles' : 'NCC(=O)',
        'upper_smiles' : 'NC',
        'lower_indices' : [0, 1, 2, 9, 10, 11],
        'residue_indices' : [3, 4, 5, 6, 12, 13, 14],
        'upper_indices' : [7, 8, 15, 16, 17, 18],
        'lower_connect_indices' : [1, 2, 3],
        'upper_connect_indices' : [5, 6, 7],
        'backbone_indices' : [0, 1, 3, 4, 5, 7, 8],
        'atoms' : [6, 6, 8, 7, 6, 6, 8, 7, 6, 1, 1, 1, 1, 1, 1, 1, 1, 1, 1],
'atom_types' : ['SP3', 'SP2', 'SP2', 'SP2', 'SP3', 'SP2', 'SP2', 'SP2', 'SP3', 'S', 'S', 'S', 'S', 'S', 'S', 'S', 'S', 'S', 'S'],
'bonds' : [[0, 1], [1, 2], [1, 3], [3, 4], [4, 5], [5, 6], [5, 7], [7, 8], [0, 9], [0, 10], [0, 11], [3, 12], [4, 13], [4, 14], [7, 15], [8, 16], [8, 17], [8, 18]],
'bond_types' : ['SINGLE', 'DOUBLE', 'SINGLE', 'SINGLE', 'SINGLE', 'DOUBLE', 'SINGLE', 'SINGLE', 'SINGLE', 'SINGLE', 'SINGLE', 'SINGLE', 'SINGLE', 'SINGLE', 'SINGLE', 'SINGLE', 'SINGLE', 'SINGLE'],
        'rotamers' : 2,
'charges' : [-0.18, 0.25, -0.38, -0.5, 0.03, 0.27, -0.39, -0.47, -0.08, 0.12, 0.1, 0.11, 0.23, 0.15, 0.11, 0.25, 0.14, 0.13, 0.11],
    },
}
```

*Boltzmann adaptive sampling:* We developed a new sampling protocol to generate the entire potential energy surface of the monomeric residues which we have called Boltzmann Adaptive Sampling (BAS). For energy evaluation in the adaptive search, we utilized the atoms-in-molecules deep neural network, AIMNet, modified with a London dispersion correction [(*18*, *19*)](https://paperpile.com/c/4WDLXM/ln5J+VF7H); this requires only atomic coordinates and atomic numbers as inputs and thus does not require complex atom typing like many molecular mechanics-based energy functions, and has accuracy comparable to DFT-based methods that are orders of magnitudes more expensive to evaluate.

The entire protocol is represented visually as a process flow diagram in Figure **S13**. The iterative process of building the Delaunay triangulation, selecting points, and evaluating the energy of the molecule is repeated until the potential energy surface of the molecule has converged. The BAS scheme utilizes Delaunay triangulation to maintain the relationship between all of the points that have been sampled. For this protocol, all points of the Delaunay triangulation are the dihedral values of conformations that have been sampled. We store the conformation and the energy of the molecule that is associated with the point. Bond torsion with many possible values, such as *phi* and *psi* of alpha amino acids, were sampled with the BAS; bond torsions with very few possible angles like amide bonds were considered as “rotamers” that were not adaptively sampled but instead they were fixed in a *cis* or *trans* configuration and evaluated in two separate runs. Likewise dihedrals that must change in concert with one another (i.e. ring flips) were treated as individual “rotamers” and evaluated in two separate runs.

The sampling protocol is initialized by sampling a sparse regular grid with 7 points per dimension (i.e. rotatable bond). This was found to sample the most common dihedral angles (-180**°**, -120**°**, -60**°**, 0**°**, 60**°**, 120**°**, 180**°**). Note that the first and last dihedral angles are identical, but Delaunay triangulation implementations that work in higher dimensions cannot account for periodicity. After the initial grid conformations are evaluated, we create the Delaunay triangulation that will guide further sampling. Every simplex of the Delaunay triangulation is evaluated with a loss function which is inspired by the Boltzmann probability (see below). The simplex with the highest loss is then chosen. Once a simplex is chosen, we select the center of the simplex with small random perturbations to avoid deterministic sampling. That point corresponds to the next set of torsions that will be evaluated. After evaluating that point by minimizing the conformation with the new dihedral angles, that point is added back into the Delaunay triangulation. This results in the selected simplex to divide into multiple new simplices. This process of identifying simplices, evaluating the centers of the simplices, and updating the Delaunay triangulation can be done individually or in batches. While performing this process one simplex at a time is optimal for sampling the simplex with the highest loss, we perform the process in small batches (<32) because the time to update the Delaunay triangulation is significant. We developed a dynamic controller that adjusted the batch size to ensure that 99% of the runtime was spent on evaluating the molecular energy. This controller was especially necessary in high dimensions because the Delaunay triangulation calculation scales poorly with number of dimensions and number of samples. If a controller is not used to adjust the batch size, the protocol will spend most of the time selecting which points to sample instead of evaluating the energy of these points. The loss function that determines which simplices to sample next is the most critical part of this protocol. We devised what we term the volume adjusted Boltzmann probability, which guides the adaptive grid to sporadically sample both small simplicities whose points are very low in relative energy, or large simplicities that have not been explored. This ensures that the adaptive grid does not continually subdivide the lowest energy simplicity which would result in narrow sampling.

Once the new points have been selected for evaluation, the monomers are constructed and subjected to minimization in AIMNet. We found that without minimization, the resulting potential energy surfaces of the molecules become sharp and discontinuous. We found that minimizing the conformation with bond torsion constraints produced smooth potential energy surfaces that qualitatively recapitulated known potential energy surfaces like alanine. It was essential to perform a full minimization on the molecule because minute changes in bond lengths and bond angles (which are not constrained in the minimization) can significantly affect the energy of a conformation. We also found that using the conformation of the nearest neighbor as the starting point before setting the major degrees of freedom and applying constraints improved the run time and convergence of the minimization trajectories. Presumably this is because the nearest neighbor point has similar perturbations to the minor degrees of freedom.

An example of a converged potential energy surface for alanine is shown in Figure **S14**. The adaptive resolution of sampling is apparent when comparing the size of the simplices in the low and high energy regions of the potential energy surface. Regions of low energy have very dense sampling and small simplices while regions of high energy have very sparse sampling and large simplices. This phenomenon emerges directly from the loss function that balances the weight of energy and volume. Because this process does not use any heuristics that are specific to any particular molecule, this process can be applied to any molecule while maintaining the exceptional sampling performance.

The key metric for performance of the new sampling protocol is the rate of convergence of the potential energy surface. The potential energy surface can be generated by linearly interpolating the Delaunay triangulation. As more points are sampled in the Delaunay triangulation, a linear interpolation of the points will become more and more accurate at predicting new interpolated points. Once enough points have been sampled, the potential energy surface for a given molecule will converge and adding more points will not affect the potential energy surface. Once the potential energy surface is converged, the energy of any conformation can be accurately predicted.

We found that the BAS identifies low-energy conformers of monomers compared to naive grid-based searches; particularly for torsionally constrained monomers like aminobutyric acid, or for monomers with many rotatable bonds (Figure **S15**). While the BAS performed well to generate potential energy surfaces for the monomers used in this study, we found that it struggles to sample the potential energy surface of molecules with more than 6 degrees of freedom. This barrier is due to the time to calculate the Delaunay triangulation and is exacerbated by the fact that higher dimensions require more sampled points in general. For example, in 6 dimensions with 1 million points, updating the Delaunay triangulation with 100 additional points is time equivalent to minimizing the energy of these 100 new points. This discrepancy between updating the triangulation and evaluating the energy of the new points on the surface becomes more dramatic in higher dimensions (i.e. when more bond torsions are sampled). For these reasons, the BAS is ideal for sampling the potential energy surfaces of monomers, but not macrocycles.

We used BAS to generate potential energy surfaces for each monomer depicted in Figure **S1**. In these calculations, each monomer surface was generated for each monomer, containing both C-terminal methylamide and C-terminal dimethylamide modifications. The latter are pre-proline and are denoted with _pp appended to the residue’s name. The potential energy surfaces are stored on disk as HDF5 files that contain datasets of the AIMNet minimized conformers of the monomer and their associated AIMNet energies (in kcal/mol) for subsequent use in hashing and macrocycle construction (see below). In the below methods, these potential energy surfaces are referred to as “conformer databases”. The following command illustrates how to generate the potential energy surface of glycine:

OMP_NUM_THREADS=1 python run_residue.py --residue AGLY --rotamer 0 --number_of_threads 4

*Sequence compatibility check*: In the below procedures, the compatibility of two monomers to construct a dimer is frequently checked before proceeding with generating hash tables or ensembles of macrocycles. Two monomers being compatible means that the monomers respect the following rules: In a dipeptide, if monomer 2 is proline_like (e.g. proline, *N*-methylated, proline-like, etc) then conformers of monomer 1 must come from the preproline conformer database of monomer 1; If monomer 2 is not proline_like, then conformers of monomer 1 must not come from the preproline conformer database of monomer 1. To facilitate this check, each monomer is ascribed two boolean properties in the residue_params dictionary; one that stores if the monomer is proline_like; the second stores if the monomer is preproline.

*Hash table construction*: Monomer hash tables are built through a three step process. First, conformers of the specified monomer are loaded into memory from the conformer databases created as described above. Conformers of the monomer can be filtered for inclusion in the hash tables by specifying an energy threshold in kcal/mol. Conformers whose energy relative to the global minimum energy for the monomer that are above this threshold are not included in the hashing. Second, coordinate frames are constructed for the terminal amides using the amide nitrogen, oxygen, and carbon atoms and the relative transformation between these coordinated frames is calculated and binned using the hash function available in the python package xbin. This is done for the *N*-terminal coordinate frame relative to the *C-*terminal coordinate frame, as well as for the *C*-terminal coordinate frame relative to the *N*-terminal coordinate frame. Third, the binned transformations, and the conformers that belong within the bins, are sorted and ultimately used to construct a getpy dictionary object, and an associated numpy array we term the key-value array. The dictionary contains as keys, the unique set of binned transformations calculated across every conformer brought into the hashing under step 1. The values that are accessed using these keys are tuples of start and stop indices that are used to select sub slices of the key-value array. Indexing the key-value array with this tuple will produce a numpy array of conformer indices that are used to access the conformers of the monomer within its conformer database that belong to the specific binned transformation (detailed below). These dictionaries and their respective key-value arrays are stored on disk as binary files for later use.

In this process, the size of the bins used for hashing is determined by two parameters, the cartesian resolution, and the origin resolution. Increasing either will discretize the 6D space into few larger bins whereas decreasing either will discretize the 6D space into many smaller bins. This can dramatically impact the quality and numbers of closures found in subsequent steps. Large bins in the 6D space will result in hashtables with fewer binned transforms that each contain many conformers. Small bins in the 6D space will result in hashtables with many binned transforms that each contain few conformers. To tune the size of these bins, we created hashtables for each monomer and varying resolutions then assessed the quality of the resulting 2-residue macrocycles found from searching these hashtables for closures. For each conformer that this search produced, we built the macrocycle by superimposing the pair of *N*-terminal amides of one monomer onto the *C*-terminal amides of the second monomer. We then measured the RMSD between these two pairs of amides in the resulting conformer (Figure **S16**). High quality closures are macrocycles where this RMSD is low. This occurs when the two conformers used to construct the macrocycle share very similar relative orientations. Low quality closures are macrocycles where this RMSD is high. This occurs when the two conformers have different relative orientations that were placed into the same bin in hashing due to large discretization of the 6D space. We found that as the size of the bins increases the resulting ensembles of macrocycles contain many conformers, however the quality of the closures is often poor. As the size of the bins decreases, the resulting ensembles of macrocycles will contain fewer conformers, but the quality of the closures increases. While poor closures can be identified in AIMNet minimizations, we focused on identifying high quality closures. As such in this study we used either 1.0 angstrom cartesian resolution with 15.0 degree origin resolution or 0.5 angstrom cartesian resolution with 10.0 degree origin resolution.

The below command demonstrates constructing hash tables of each monomer within HDF5_DIR to include conformers no more than 6 kcal/mol above the global minimum using 1.0 angstrom cartesian and 15.0 degree origin binning resolutions, which will be save in HASH_DIR/<kT>kcal_<cart_resl>A_<ori_resl>deg/:

python make_monomer_NC-CN_hash.py -kT 6.0 -cart_resl 1.0 -ori_resl 15.0 -hash_dir <full path to HASH_DIR> -hdf5_dir <full path to HDF5_DIR>

Hash tables for dimers are constructed in a nearly identical process. First, conformers for two monomers, monomer 1 and monomer 2, are loaded into memory after assessing their sequence compatibility. Second, each pair of monomer 1 and monomer 2 conformers is aligned to construct a dimer. In this alignment, the *C-*terminal amide of monomer 1 is aligned to the *N*-terminal amide of monomer 2. The relative transformation between coordinate frames of the terminal amides (*N*-termi of monomer 1 and *C*-termini of monomer 2) are calculated and binned. The resulting getpy dictionary and associated key-value array is stored on disk for future use. Here, the key-value array stores two indices, instead of one index. The first is used to identify the conformer of monomer 1 in the dimer, the second is used to identify the conformer of monomer 2 in the dimer that belongs to the binned transform in question. The following command demonstrates constructing hash tables of a proline-alanine dipeptide:

python make_dimer_NC-CN_hash.py -m1 PRO -m2 ALA -kT 1.0 -cart_resl 0.5 -ori_resl 10.0 -hash_dir <full path to HASH_DIR> -hdf5_dir <full path to HDF5_dir>

Hashtables that contain dimers with hydrogen bonding interaction are constructed by first identifying conformations of the dimer that contain hydrogen bonds, and only using those conformers in hashing. Hydrogen bonds are identified with a rapid geometric score term. This score term ranges between 0 and 1 with higher numbers producing more linear hydrogen bonds between amides. In this study we generated hydrogen bond-containing hashtables that scored above 0.75 with this term, and contained conformers no higher than 4 kcal/mol above the global minimum. The below command demonstrates this for the same proline alanine dipeptide, ensuring that no conformers that contain cis amides are present (unless the monomer is proline_like) and that the quality of the hbond interaction is at least greater than 0.75:

python make_dimer_hbond_NC-CN_hash.py -m1 B3ALA -m2 ACBEN2 -kT 4.0 -cart_resl 0.5 -ori_resl 10.0 --filter_out_cis --hbond_threshold 0.5 -hash_dir <full path to HASH_DIR> -hdf5_dir <full path to HDF5_dir>

*Chemotype and sequence alphabetization*: Each monomer is assigned a single character that defines the number and hybridization of atoms along the backbone, starting from the *N*-terminal amide nitrogen, and ending at the *C*-terminal amide carbon. These single characters, or chemotypes, represent the first layer of alphabetization. Additionally, each monomer is assigned a priority based on its arbitrary location in a list of all monomers. This priority represents the second layer of alphabetization. When combined, the first and second layers of alphabetization generate an offset that is used to permute the input sequence into the lowest priority alphabetized chemotype. This ensures that a single sequence of monomers, regardless of their circular permutation, generates a single output macrocycle during ensemble generation. The methods to accomplish this alphabetization are in coarse_cluster.py

The first layer of alphabetization is used to contract circularly permuted backbones into the same chemotype. For example, the chemotypes aaab, aaba, abaa, and baaa are all the same chemotype, just permuted circularly. Given an input sequence of monomers we assign the sequence a chemotype, then permute that sequence into the circular permutation that is lowest priority when alphabetized. In the aforementioned example, aaab, aaba, abaa, and baaa will all be permuted into aaab.

The second layer of alphabetization is used to ensure that an input sequence of monomers, regardless of circular permutation, only generates a single macrocyclic sequence. For chemotypes that contain some element of circular symmetry (e.g. aaaa, abab, etc) there exists degenerate circular permutations. For example, acac can be permuted into the four following chemotypes: acac, caca, acac, and caca. By chemotype alphabetization, the first and third permutation in this are the same. In such chases, the monomer priorities are used to select between these degenerate chemotypes.

*Ensemble generation*: Ensembles are generated in a four-step procedure. First the requested hash tables (as getpy dictionaries) are loaded into memory. Second, any shared keys between both hashtables are identified and the conformer indices belonging to these shared bins are extracted from their respective key-value arrays. Third, all combinations of conformer indices of the N-to-C shared bin are combined with all combinations of conformer indices of the C-to-N shared bin. This procedure produces two dimensional numpy arrays that we term IJKL. The IJKL arrays are (M_conformers x i_resdiues) where M_conformers is the number of macrocycle conformers within the ensemble, and i_residues are the number of monomers in that macrocycle. For example, the 100 membered ensemble of the 3-residue cycle aaa-AGLY-AGLY-AGLY will have an IJKL array that is (100, 3), and IJKL[0,:] are the indices of the monomer conformers used to construct the 0th conformer of the macrocycle. IJKL[0,0] is the first conformer index of the first glycine monomer of the macrocycle, IJKL[0,1] is the first conformer index of the second glycine monomer of the macrocycle, etc. Third, the second dimension of the IJKL array is permuted into the lowest priority sequence according to the alphabetization process detailed above. The permuted IJKL array is finally saved to disk within an HDF5 file that is named according to the lowest priority sequence for the macrocycle.

The following commands demonstrate ways to generate 3 and 4 residue macrocycles. In all of these commands, note that the cartesian resolution and origin resolution must be specified and match those used to construct the hash tables. It is also important to segregate the dimer and monomer hashtables into separate HASH_DIR. All of these commands will attempt to generate ensembles and output them to CSV_DIR/SUB_DIR. Saving the ensembles into a handful of subdirectories helps ensure the file quota limits are not reached in large scale sampling. Possible write collisions that can arise due to circular permutations in sequence are further avoided by appending a unique string to the output ensemble name.

The following command demonstrates searching for ensembles of every 3-residue macrocycle that contains a proline-alanine dipeptide at 1.0 kcal/mol that can be constructed from monomer hashtables in <HASH_DIR>:

python build_3mers_ensemble.py -m1 PRO -m2 ALA -cart_resl 0.5 -ori_resl 10.0 -kT 1.0 -hash_dir <full path to monomer HASH_DIR> -csv_dir <full path to where output ensembles will be saved> -sub_dir <subdirectory within CSV_DIR> -sub_file <unique string> -hdf5_dir <full path to HDF5_dir containing conformer databases>

The following command demonstrates generating ensembles for every possible 4-residue macrocycle that can be constructed from dimer hashtables in <HASH_DIR> that close the proline alanine dipeptide:

python build_4mers_ensemble.py -m1 PRO -m2 ALA -cart_resl 0.5 -ori_resl 10.0 -hash_dir <full path to HASH_DIR> -csv_dir <full path to where output ensembles will be saved> -sub_dir <subdirectory within CSV_DIR> -sub_file <unique string>

The following command demonstrates attempting to generate an ensemble for only the macrocycle aaaa-ALA-ALA-ALA_pp-PRO:

python build_4mers_ensemble.py -m1 PRO -m2 ALA -m3 ALA -m4 ALA_pp -cart_resl 0.5 -ori_resl 10.0 -hash_dir <full path to HASH_DIR> -csv_dir <full path to where output ensembles will be saved> -sub_dir <subdirectory within CSV_DIR> -sub_file <unique string>

After generating these ensembles within subdirectories with unique names appended to the ensembles, the following command will combine the unique conformers of every sequence within a chemotype split across these separate ensembles into a single HDF5 file for each chemotype:

python combine_seperated_ensembles.py <CHEMOTYPE> <full path to WORK_DIR>

Note that WORK_DIR here should contain CSV_DIR, and a directory named HDF5. The combined ensembles will be saved in WORK_DIR/HDF5/CHEMOTYPE/.

*Generation of 3D coordinates of macrocycles from ensembles stored in HDF5s*: Prior to AIMNet minimizations or any other analysis, the IJKL array that stores the ensemble is converted into a (M_conformers x N_atoms x 3) array of atomic coordinates in addition to a (N_atoms, ) array of atomic numbers through a process we term building. We perform building from the ensemble IJKL arrays in memory immediately prior to any analysis / minimization to reduce the amount of space required to store the ensembles on disk. The below paragraphs details the procedure for building a 4-residue macrocycle.

First, all of the conformers from monomers in the given sequence are loaded from their respective conformer databases as (X_conformers, Y_atoms, 3) numpy arrays of atomic coordinates. The IJKL array is then used to index these atomic coordinates on the X_conformers axis, for each monomer. For a four residue macrocycle, this will produce four masked arrays, one for each monomer in the sequence. These arrays are (M_conformers, Y_atoms, 3) in shape, where M matches the number of conformers in the macrocycle ensemble and Y is the number of atoms in that specific monomer. Second, these masked arrays go through an initial build cycle where the *N-*terminal amide of monomer i+1 is aligned to the *C*-terminal amide of monomer i. This loop runs from i=1 to i=3, for a four residue macrocycle. Once this 3 residue fragment is constructed, the *N-*terminal amide of the final monomer is aligned to the C-terminal amide of the third monomer while simultaneously aligning the *C-*terminal amide of the final monomer to the *N*-terminal amide of the first monomer.

Third, each monomer is subsequently realigned into the macrocycle such that the *C*-terminal amide of monomer i is aligned the the *N-*terminal amide of monomer i+1 while simultaneously aligning the *N*-terminal amide of monomer i with the *C*-terminal amide of monomer i-1. This process cycles through a while loop that tracks the average RMSD between the amides that are being spliced together. This cycle completes once the average RMSD is no longer decreasing or a max number of cycles has been reached. Typically, we allowed no more than 200 cycles in this build loop.

At the end of this cycle, the four aligned (M_conformers, Y_atoms, 3) XYZ arrays are spliced together to construct the final (M_conformers, N_atoms, 3) XYZ array of the macrocycle, which we term the macrocycle_xyz array, in addition to the (N_atoms, ) array of atomic coordinate, which we term the macrocycle_atoms array. These arrays can be used in subsequent calculations. The methods that accomplish these steps are implemented in utils_build.py.

*Torsion-bin clustering*: Torsion bin clustering is accomplished through the following steps. First, the 3D coordinates of every conformer in an ensemble are built in memory as described above. The resulting macrocycle_xyz array is masked to only contain the XYZ coordinates of the backbone atoms. These XYZ coordinates are converted into internal coordinates which produce an (M_confomers, N_backbone_atoms, 3) array of bond length, bond angle, and bond torsions that we term the dofs array. The dihedral angles are extracted from the dofs array and converted from an angle into an integer using the following equation:

bins = ((np.around(dofs[:,:,-1] / torsion_resolution) + 3) % 6 - 3).astype(np.int8)

Where the torsion_resolution is set to 60 degrees. These bins are then saved into a new HDF5 for subsequent analysis. The following command is used to accomplish this torsion binning:

python analyze_macrocycle_torsions.py -i <full path to ensemble> --write_cluster_hdf5 -out_dir <path to save output HDF5> -pdb_dir <path to save PDB files of cluster representatives> -hdf5_dir <path to conformer databases>

*Principal moment of inertia analysis*: The principal moments of inertia ratios for torsion clusters of several chemotypes were calculated as described [(*20*)](https://paperpile.com/c/4WDLXM/1elb). We implemented this calculation within the analyze_macrocycle_torsions.py script. The following command adds the two PMI ratios into the output HDF5 from torsion clustering as datasets labeled I1_I3 and I2_I3:

python analyze_macrocycle_torsions.py --moi -i <full path to ensemble> --write_cluster_hdf5 -out_dir <path to save output HDF5> -pdb_dir <path to save PDB files of cluster representatives> -hdf5_dir <path to conformer databases>

In our analysis, we only use the backbone heavy atoms to determine the two PMI ratios.

*AIMNet Minimizations and Pnear calculations*: Ensembles of macrocycles were minimized in the AIMNet score function modified with the dftd4 correction using the ase python package using the BFGS optimizer. Each macrocycle conformer is constrained using the FixInternals constraints to ensure no chemical reactions occurred during minimization. Geometry optimization was run for no more than 100 steps, or until the max force did not exceed 0.005 eV/A. In these optimizations, the maxstep was set to 0.5 angstroms. This minimization scheme typically takes about 1-3 minutes on a CPU per macrocycle conformer to complete, depending on the number of atoms in the macrocycle.

We implemented this minimization in the minimze_macrocyle_ensemble.py script. This script first builds the 3D coordinates of the macrocycle from the input ensemble as described above, then subjects the conformers to minimization. This script enables parallelizing this minimization across many CPU cores using the flag -j. In our testing, each parallel job requires approximately 3 GB of RAM. After all conformers have been minimized, the lowest energy conformer from the ensemble is identified. The backbone atoms of all other conformers are aligned to the backbone atoms of the low energy conformer. The RMSD of these alignments are calculated. The RMSD and AIMNet energies are used to construct an energy landscape like those depicted in Figure **1B** and **S6**. The Pnear value for the landscape is calculated with kT=0.62 and lamda=0.3 angstroms as previously described [(*21*)](https://paperpile.com/c/4WDLXM/NXLr). The below command demonstrates minimizing a random 1000 conformers from an ensemble. This command parallelizes this minimization across 20 CPU cores:

OMP_NUM_THREADS=1 python minimize_macrocycle_ensemble.py -i <full path to ensemble> --endpoint_only --limit 1000 -j 20 --save_hdf5 -hdf5_dir <path for conformer databases> -pdb_dir <path to PDB_DIR> -png_dir <path to PNG_DIR>

This will produce a SVG file of the energy landscape in PNG_DIR, a PDB file that contains up to the 100 lowest energy conformers of the macrocycle in PDB_DIR, and a HDF5 file that contains the XYZs of the minimized ensemble, their RMSDs to the lowest energy conformer within the ensemble, and their AIMNet minimized energies saved into PDB_DIR. We manually inspected the lowest energy conformers macrocycles if the Pnear of their respective energy landscape was greater than 0.75.

*Sequence design of hydrogen bonding macrocycles*: We identified specific sequences capable of forming long rang hydrogen bonds using an augmented pipeline from that described above. We first identified a set of base residues within each chemotype. We then generated ensembles for all possible 4-residue macrocycles that contain combinations of only these base residues by searching for closures amongst the hydrogen bonds containing hash tables. Such ensembles are created by adding the –-only_base_residues flag to build_4mers_ensembles.py. We then clustered the resulting ensembles using the analyze_macrocycle_torsions.py script to produce pdb files containing a single representative of each torsion bin sampled in the ensemble. We then used the analyze_compatible_sequences.py script to identify possible low-energy sequences for each of the torsion-bin representatives. We then generate ensembles for each of the candidate low-energy sequences, and subject those ensembles to minimization as described above.

Candidate low-energy sequences are identified in the analyze_compatible_sequences.py script through 1-body sidechain packing algorithm (Figure **S17**). First, we identify possible side chain substitutions that are possible at each position or the macrocycle from the sequence of the base residue macrocycle (e.g. mutating glycine to alanine, phenylalanine, etc). We then rank-order these modifications based on their compatibility with the backbone torsion angles of the torsion-bin representative being processed. We query the potential energy surface of each possible modification at each position in the macrocycle to identify the relative energy of the lowest energy rotamer possible for each modification given the backbone torsion angles of the base residue in the macrocycle. This process allows identifying the monomers most likely to accommodate the torsion angles present in the torsion-bin representative. This is done for each position in the macrocycle being processed. This process results in a list of sequences, each of which is composed of the ideal modification of the base residue in each of the torsion-bin representatives. We then used build_4mers_ensembles.py to construct ensembles of just those sequences. We found that this analysis produced sequences that, when minimized, reduced the occurrence of non-ideal torsion angles such as L-amino acids being placed at positions with positive phi torsion angles.

The below command is used to produce lists of candidate sequences for ensemble generation:

python analyze_compatible_sequences.py -i <full path to pdb from torsion binning> -hdf5_dir <path for conformer databases> -out_dir <path to save output lists>

We also used this process to identify at each position the 3 lowest energy monomers, and created candidate sequences of each combination at each position by adding the -max_monomers 3 flag.

We minimized approximately 8280 hydrogen bond-containing macrocycles resulting from the above sequence design process. To prioritize macrocycles from this subset for manual inspection and synthesis, we constructed a simple linear regression model that predicts the AIMNet energy of a macrocycle given its sequence composition (Figure **S18**). We constructed a matrix wherein each column is associated to a specific monomer and each row is associated to a macrocyclic sequence. For a given macrocycle, this matrix counts the number of specific monomers in each sequence. We then constructed a vector wherein each row contains the AIMNet energy of the low energy conformer of the macrocycle. We then used the sklearn python package to train weights associated with each monomer in the matrix using the LinearRegression model. We found that this model predicted the AIMNet energy of the low energy conformer of a given macrocycle within 5 energy units. We then prioritized macrocycles for inspection if the ZScore (calculated from the minimized AIMNet energy + the predicted AIMNet energy) was less than -2. This process identified macrocycles whose AIMNet energy is much more negative (i.e. favorable) than anticipated, given the sequence of the macrocycle.

*Target preparation*: The X-ray crystallographic structure of a cyclic peptide des4.3.1 (PDB 6WSJ) bound to HDAC6 was used as the starting point for the design of the small macrocycles that inhibit HDACs. In PyMOL, all waters, crystallization buffer components, and metals were deleted from the model. Additionally, all residues in des4.3.1, except the d-arginine at position 4 and the unnatural amino acid SHA at position 5, were deleted from the model. These modifications were saved to a new PDB file containing HDAC6 in chain A, and a dipeptide dARG-SHA in chain B. In a text editor, the sidechain atoms of the d-arginine and the C-alpha proton of the d-arginine were deleted from the model. The residue identity of the remaining atoms belonging to d-arginine were changed to either ‘DPR’, ‘PRO’, or ‘ALA’. These modifications result in three separate PDB files, with HDAC6 in chain A, and either dPRO-SHA, PRO-SHA, or dALA-SHA in chain B. These three models serve as the input to the hash closure steps detailed below.

The X-ray crystallographic structure of a linear *c*-terminal prosequence (PDB 5B6O) bound to SARS-CoV main protease was used as the starting point for the design of the small macrocycles that inhibit SARS-CoV-2 MPro. In PyMOL, all waters, crystallization buffer components, and metals were deleted from the model. Additionally, all residues in the linear prosequence, except the phenylalanine-glutamine dipeptide, were deleted from the model. These modifications were saved to a new PDB file containing MPro in chain A, and a dipeptide PHE-GLN in chain B. This model serves as the input to the hash closure steps detailed below.

*Macrocycle design*: The dipeptides in the above models are modified using a PyRosetta script with N-terminal acetyl amide and C-terminal methyl amide patches. The N-terminal phi torsion angle, and the C-terminal psi torsion angle of the modified dipeptides are perturbed to bring the terminal amides into a hydrogen-bonding geometry. The relative transform between the plane of the terminal amides is then calculated and hashed in the C-to-N direction. Closures are found by then searching for common binned transforms between a database of N-to-C hashtables and the C-to-N hashtable of the dipeptide bound to the target of interest. Full atom representations of any conformer found to close the dipeptide in this search are constructed in context of the target.. Clashes between these conformers and the target are then evaluated in a voxel-based clash check. Conformers with less than 5 clashing voxels were then saved to disk as PDB files for subsequent analysis. This pipeline is implemented in several pyrosetta scripts. The below bash command is used to launch this pipeline to identify candidate Mpro-binding macrocycles:

sh Mpro_example_cyclize.sh

Each output PDB file of a macrocycle found in the hash closure step is parameterized as a ligand for use in the Rosetta software suite using mol2genparams.py, included with Rosetta. Each macrocycle is then evaluated using GALigandDock within the Rosetta software suite, using the below commands:

$ROSETTABIN/rosetta_scripts -mute all \

-gen_potential \

-beta_cart \

-no_autogen_cart_improper \

-parser:protocol dock_flex.xml \

-s DUMMY.pdb \

-in:file:extra_res_fa DUMMY.params LG1.params \

-parser:script_vars ligand=LG1 \

-nstruct 5 \

-overwrite \

Where “dock.xml” is as below:

```
<ROSETTASCRIPTS>
  <SCOREFXNS>
	<ScoreFunction name="genpot_soft" weights="beta_cart">
  	<Reweight scoretype="fa_rep" weight="0.2"/>
	</ScoreFunction>
	<ScoreFunction name="genpot" weights="beta_cart">
	</ScoreFunction>
  </SCOREFXNS>
  <RESIDUE_SELECTORS>
   	 <Index name="SelLig" resnums="1" reverse="true" />
	<Not name="not_lig" selector="SelLig"/>
   	 <Neighborhood name="Lig_neighbors" selector="SelLig" distance="10.0" />
	<And name="binding_surface" selectors="Lig_neighbors,not_lig"/>
	<ResidueName name="metal" residue_names="ZN"/>
  </RESIDUE_SELECTORS>
  <MOVERS>
	<GALigandDock
    	name="dock"
    	scorefxn="genpot_soft"
    	scorefxn_relax="genpot"
    	runmode="dockflex"
    	premin_ligand="1"
    	padding="2.5"
    	multiple_ligands="%%ligid%%"
    	estimate_dG="1" />
  </MOVERS>
  <PROTOCOLS>
	<Add mover="dock"/>
  </PROTOCOLS>
  <OUTPUT scorefxn="genpot"/>
</ROSETTASCRIPTS>
```

For HDAC6-based designs, the input file “DUMMY.pdb” was prepared from the X-ray crystallographic structure of a cyclic peptide des4.3.1 (PDB 6WSJ) bound to HDAC6. This model was modified to contain HDAC6 first, the Zn atom second, and the coordinates of a DUMMY ligand placed near the active site last. This ligand is replaced with the ligand in “LG1.params” at runtime. For MPro-based designs, the input file “DUMMY.pdb” was prepared from the X-ray crystallographic structure of a covalent inhibitor bound to SARS-CoV-2 MPro (PDB 6LU7). In PyMOL, all waters, crystallization buffer components, and metals were deleted from the model. MPro was placed in the first chain while a DUMMY ligand was placed near the active site last. This ligand is replaced with the ligand in “LG1.params” at runtime.

The models of the designed macrocycle:target complexes were sorted by the most favorable GALigandDock reported dG of binding and manually inspected. Approximately 200 models were inspected per target. Macrocycles were prioritized for synthesis if the lowest energy docked model closely aligned with their respective outputs form the hash closure (i.e. those macrocycles that “forward docked”), and made additional contacts with the target beyond those made by the starting dipeptides. Ultimately, 6 such macrocycles were chosen for chemical synthesis and testing against MPro, whereas 11 such macrocycles were selected for synthesis and testing against HDAC6.

*In silico sidechain mutant library generation in ICM:* The above protocol identified aabi-MPRO-1 as a modest inhibitor of MPro. An *in silico* library of *ca*. 600 macrocycles were generated from the GALigandDock-generated model of PJS-MPRO-1 bound to MPro using custom scripts implemented in ICM software suite. These scripts produce all combinations of 3D models of homologues of aabi-MPRO-1 where the glutamine, phenylalanine, and B3-phenylalanine are mutated to one of 3, 21, or 10 modifications, respectively. This small *in silico* library was re-docked into MPro using GALigandDock as described above. Models of the homologues:MPro complexes were sorted as described above. Ultimately, 27 homologues were chosen for chemical synthesis and testing. The below command is used to conduct the combinatorial generation of sidechain mutants of aabi-MPRO-1, after saving to coordinates of the model to an SDF file:

$ICMHOME/icm64 modify.icm <path/to/models/to/modify/as/.sdf>

where “modify.icm” is as below:

```
l_commands = no
l_info = no
l_warn = no

HELP = """
  Usage> $P <input.sdf> [<output.sdf>]
"""

if (Getarg(help)) quit HELP

call _macro
call _ligedit

LIGAND.minimizeAfterModifyGroup = no
LIGAND.chargeAfterModifyGroup = no
TOOLS.tsWeight=LIGAND.tsWeight
LIGAND.energyDisplayState=no

icIndexForce = no

# REPLACE it with actual input
S_files = Getarg(list delete)

if !(Nof( S_files ) == 1 | Nof( S_files ) == 2) quit HELP

s_infile = S_files[1]
if (Nof(S_files) == 2) then
  s_outfile = S_files[2]
else
  s_outfile = Path(s_infile) + Name(s_infile) + "_out.sdf"
endif

s_ixfile = Path(s_infile)+Name(s_infile)+".inx"
if ( !Exist(s_ixfile) | icIndexForce | Newer(s_infile s_ixfile) ) write index mol s_infile s_ixfile
s_ixfile

read index s_ixfile name="ix"
nofComp = Nof( ix )

delete system s_outfile
#read table mol s_infile name="chem"


# method for all combinations
function getAllComb( i_nmatch i_nsubst )
  res = Array()
  I_out = Iarray(i_nmatch, 0 )
  while yes
     I_out[1] = I_out[1] + 1
     for i=1,i_nmatch
       if (I_out[i] == i_nsubst+1) then
          if (i == i_nmatch) break
          I_out[i] = 0
          I_out[i+1] = I_out[i+1] + 1
       endif
     endfor
     if (i == i_nmatch) break
     res[Nof(res)+1] = I_out
  endwhile
  return res
endfunction


c_repl = Array()

# phenylalanine based stubs
c_repl[Nof(c_repl)+1] = Collection( "what" "[c;D2]1[c;D2][c;D2][c;D2][c;D2][c;D3]1[$(CC(C=O)[N;D2])]" \
                                    "with" { "[C*]c1ccc(F)cc1", "[C*]C1CCCCC1", "[C*]c1ccc(C#N)cc1", "[C*]c1cScc1", "[C*]c1ccc(I)cc1", "[C*]c1ccc(Br)cc1", "[C*]c1ccc(Cl)cc1", "[C*]c1cc(Cl)ccc1", "[C*]c1ccc(Cl)cc1", "[C*]c1c(F)c(F)c(F)c(F)c1(F)",  "[C*]c1ccc(C)cc1", "[C*]c1c(C)cccc1", "[C*]c1cc(C)ccc1", "[C*]c1c(C)cc(C)cc1", "[C*]c1ccc(C(C)(C)C)cc1", "[C*]c1cc(OC)ccc1", "[C*]c1ccc(OC)cc1", "[C*]c1cc(F)cc(F)c1", "[C*]c1c(C(F)(F)F)cccc1", "[C*]c1cc(C(F)(F)F)ccc1", "[C*]c1ccc(C(F)(F)F)cc1" } )

# Aminobutyiuc acid based stubs
c_repl[Nof(c_repl)+1] = Collection( "what" "[C;D1][$([C;D2]C(C=O)[N;D2])]" \
                                    "with" { "[C*]CC(=O)N", "[C*]c1cccnc1", "[C*]S(=O)(=O)C", "[C*]C4=CNC5=NC=CC=C54"} )


# Beta_phenylalanine based stubs
c_repl[Nof(c_repl)+1] = Collection( "what" "[c;D2]1[c;D2][c;D2][c;D2][c;D2][c;D3]1[$(CC(CC=O)[N;D2])]" \
                                    "with" { "[C*]c1ccc(Cl)cc1", "[C*]c1ccc(F)cc1", "[C*]c1ccc(I)cc1", "[C*]c1ccc(Br)cc1", "[C*]c1cc(Cl)ccc1", "[C*]c1cc(C)ccc1", "[C*]c1c(Cl)cccc1", "[C*]c1cc(Cl)c(Cl)cc1", "[C*]c1cc(F)c(F)cc1(F)", "[C*]c1c(F)c(F)c(F)c(F)c1(F)" } )

n_out = 0

for ichem=1,Nof(ix)
    read mol table ix[ichem] name="chem"
    read mol chem.mol[1] name="pep" delete
    print "Processing: " ichem " out of " nofComp
    build hydrogen
    set type mmff
    convert auto
    delete chem_out
    for i=1,Nof(c_repl)
        s_what = c_repl[i,"what"]
        S_with = c_repl[i,"with"]

        find chemical all s_what a_
        as_match = as_out
        n_subst = Nof(S_with)

        if (Nof(as_match) > 0) then
            n_match = Nof( Chemical( as_match exact ) molecule )[1]
            n_comb = Integer( Power( 2  n_match ) )-2
            for j=1,n_match
                for x=1,n_subst
                    copy a_ "tmpcopy" delete selection
                    set object a_tmpcopy.
                    set field Select( a_// & as_match molecule j ) name="subst_" + j
                    as_graph = Select( a_// "subst_" + j )
                    s_selfGroup = ""
                    e3dPrepareGroupReplace as_graph s_selfGroup
                    modifyGroupSmiles as_graph S_with[x] yes yes no
                    if (Nof(vs_out) > 0) then
                        ssearch  mute vs_out "vw,14,to,hb,ts" selftether=as3_out | a_//!vt* [1]
                    endif
                    nsubst += 1
                    if (nsubst > 0) then
                        add chem_out chem   # copy original SDF fields
                        chem_out.mol[ Nof(chem_out) ] = Chemical( a_ exact hydrogen )
                    endif
                    delete a_tmpcopy.
                endfor
            endfor
        endif
    endfor
    delete a_

    if (Nof(chem_out)) then
        write table mol append chem_out s_outfile
        n_out += Nof(chem_out)
    endif
endfor

printf " Info> %d molecules stored in %s\n", n_out, s_outfile

quit
```

*MCL1 virtual screening workflow*

*Scaffold preparation:* For the small library, we selected *ca*. 140,000 low-energy decoys from AIMNet-minimized ensembles that were generated from the hydrogen-bond containing hashtables to populate our scaffold set. For the large library, we selected approximately 14 million torsion-bin representatives from myriad ensembles generated from the locally encoded hash tables. Each scaffold set was stored as SDF files and used as input to ICM scripts that performed the combinatorial mutations to each scaffold structure.

*Library enumeration*: The small and large libraries were generated using similar ICM scripts as those used for the *in silico* sidechain mutant library described above. Each scaffold was processed with an ICM script that mutated phenylalanines, b3-phenylalanines, and sarcosine to ncAAs that bear a dichlorobenzene side-chain. We refer to this mutation as the stub in the below text:

$ICMHOME/icm64 mutate_dichloro.icm <path/to/models/to/modify/as/.sdf>

where “mutate_dichloro.icm” is as below:

```
l_commands = no
l_info = no
l_warn = no

HELP = """
  Usage> $P <input.sdf> [<output.sdf>]
"""

if (Getarg(help)) quit HELP

call _macro
call _ligedit

LIGAND.minimizeAfterModifyGroup = no
LIGAND.chargeAfterModifyGroup = no
TOOLS.tsWeight=LIGAND.tsWeight
LIGAND.energyDisplayState=no

icIndexForce = no

# REPLACE it with actual input
S_files = Getarg(list delete)

if !(Nof( S_files ) == 1 | Nof( S_files ) == 2) quit HELP

s_infile = S_files[1]
if (Nof(S_files) == 2) then
  s_outfile = S_files[2]
else
  s_outfile = Path(s_infile) + Name(s_infile) + "_out.sdf"
endif

s_ixfile = Path(s_infile)+Name(s_infile)+".inx"
if ( !Exist(s_ixfile) | icIndexForce | Newer(s_infile s_ixfile) ) write index mol s_infile s_ixfile
s_ixfile

read index s_ixfile name="ix"
nofComp = Nof( ix )

delete system s_outfile
#read table mol s_infile name="chem"


# method for all combinations
function getAllComb( i_nmatch i_nsubst )
  res = Array()
  I_out = Iarray(i_nmatch, 0 )
  while yes
 	I_out[1] = I_out[1] + 1
 	for i=1,i_nmatch
   	if (I_out[i] == i_nsubst+1) then
      	if (i == i_nmatch) break
      	I_out[i] = 0
      	I_out[i+1] = I_out[i+1] + 1
   	endif
 	endfor
 	if (i == i_nmatch) break
 	res[Nof(res)+1] = I_out
  endwhile
  return res
endfunction


c_repl = Array()


# phenylalanine based stubs
c_repl[Nof(c_repl)+1] = Collection( "what" "[c;D2]1[c;D2][c;D2][c;D2][c;D2][c;D3]1[$(CC(C=O)[N;D2])]" \
                                	"with" { "[C*]c1cc(Cl)c(Cl)cc1",} )
## N_methyl phenylalanine based stubs
c_repl[Nof(c_repl)+1] = Collection( "what" "[c;D2]1[c;D2][c;D2][c;D2][c;D2][c;D3]1[$(CC(C=O)[N;D3])]" \
                                	"with" { "[C*]c1cc(Cl)c(Cl)cc1",} )

# Sarcosine based Stubs
c_repl[Nof(c_repl)+1] = Collection( "what" "[$([C;D1]N[C;D2][C;D3](=O))]" \
                                	"with" { "[C*]c1cc(Cl)c(Cl)cc1", "[C*]Cc1cc(Cl)c(Cl)cc1",} )

# Beta_phenylalanine based stubs
c_repl[Nof(c_repl)+1] = Collection( "what" "[c;D2]1[c;D2][c;D2][c;D2][c;D2][c;D3]1[$(CC(CC=O)[N;D2])]" \
                                	"with" {"[C*]c1cc(Cl)c(Cl)cc1",} )

n_out = 0

for ichem=1,Nof(ix)
	# read next entry in 3D
	read mol table ix[ichem] name="chem"
	read mol chem.mol[1] name="pep" delete
	print "Processing: " ichem " out of " nofComp
	# convert to ICM
	build hydrogen
	set type mmff
	convert auto
	delete chem_out   # results for this row

	### Loop over the number of things in the c_repl array
	for i=1,Nof(c_repl)

    	s_what = c_repl[i,"what"]
    	S_with = c_repl[i,"with"]

    	#print s_what S_with Nof(c_repl) Nof(S_with)

    	find chemical all s_what a_   # search for all pattern
    	as_match = as_out

    	#print s_what, "number_of_matches=" Nof(as_match)
    	n_subst = Nof(S_with)

    	if (Nof(as_match) > 0) then

        	#print s_what
        	as_match = as_match | ( Next (as_match bond ) & a_//h* )# from euguene
        	n_match = Nof( Chemical( as_match exact ) molecule )[1]	# number of matches 1,2,3...
        	n_comb = Integer( Power( 2  n_match ) )-2

        	##### Call the function here to get all combinations...
        	#all_comb = getAllComb( n_match Nof(S_with) )

        	#print "n_match=" n_match, "n_comb=" n_comb, "n_comb_full=" n_comb_full
        	#all_comb, Nof(all_comb), n_comb

        	#### Loop over all combinations...
        	for j=1,n_match

            	for x=1,n_subst

                	#print all_comb[j]

                	copy a_ "tmpcopy" delete selection
                	set object a_tmpcopy.

                	# select the substituion
                	set field Select( a_// & as_match molecule j ) name="subst_" + j

                	# make subsitution wiht bitwise logic
                	as_graph = Select( a_// "subst_" + j )
                	s_selfGroup = ""
                	e3dPrepareGroupReplace as_graph s_selfGroup
                	modifyGroupSmiles as_graph S_with[x] yes yes no  ###  <- This will likely need to be changed if there are mutliple sinlge matches to a substrucutre to be repalced
                	# optmize new group variables
                	if (Nof(vs_out) > 0) then
                    	ssearch  mute vs_out "vw,14,to,hb,ts" selftether=as3_out | a_//!vt* [1]
                	endif
                	nsubst += 1
                	if (nsubst > 0) then
                    	add chem_out chem   # copy original SDF fields
                    	chem_out.mol[ Nof(chem_out) ] = Chemical( a_ exact hydrogen )
                	endif
                	delete a_tmpcopy.
            	endfor
        	endfor
    	endif
	endfor
	delete a_

	if (Nof(chem_out)) then
    	write table mol append chem_out s_outfile
    	n_out += Nof(chem_out)
	endif
endfor

printf " Info> %d molecules stored in %s\n", n_out, s_outfile

quit
```

The resulting output SDF files were again processed by a separate ICM script that combinatorially mutates a subset of the remaining side chains to a host of other ncAAs. The locations of each mutation was chosen to minimize perturbation of the input scaffolds. For example, sarcosines are mutated to myriad peptoids, phenylalanines are mutated to various ncAA phenylalanine variants, valines are mutated to myriad beta-branched amino acids etc.

$ICMHOME/icm64 mutate.icm <path/to/models/to/modify/as/.sdf>

where “mutate.icm” is as below:

```
l_commands = no
l_info = no
l_warn = no

HELP = """
  Usage> $P <input.sdf> [<output.sdf>]
"""

if (Getarg(help)) quit HELP

call _macro
call _ligedit

LIGAND.minimizeAfterModifyGroup = no
LIGAND.chargeAfterModifyGroup = no
TOOLS.tsWeight=LIGAND.tsWeight
LIGAND.energyDisplayState=no

icIndexForce = no

# REPLACE it with actual input
S_files = Getarg(list delete)

if !(Nof( S_files ) == 1 | Nof( S_files ) == 2) quit HELP

s_infile = S_files[1]
if (Nof(S_files) == 2) then
  s_outfile = S_files[2]
else
  s_outfile = Path(s_infile) + Name(s_infile) + "_out.sdf"
endif

s_ixfile = Path(s_infile)+Name(s_infile)+".inx"
if ( !Exist(s_ixfile) | icIndexForce | Newer(s_infile s_ixfile) ) write index mol s_infile s_ixfile
s_ixfile

read index s_ixfile name="ix"
nofComp = Nof( ix )

delete system s_outfile
#read table mol s_infile name="chem"


# method for all combinations
function getAllComb( i_nmatch i_nsubst )
  res = Array()
  I_out = Iarray(i_nmatch, 0 )
  while yes
 	I_out[1] = I_out[1] + 1
 	for i=1,i_nmatch
   	if (I_out[i] == i_nsubst+1) then
      	if (i == i_nmatch) break
      	I_out[i] = 0
      	I_out[i+1] = I_out[i+1] + 1
   	endif
 	endfor
 	if (i == i_nmatch) break
 	res[Nof(res)+1] = I_out
  endwhile
  return res
endfunction


c_repl = Array()

# ortho: [c*]c1ccc(X)cc1
# meta : [c*]c1cc(X)ccc1
# para : [c*]c1c(X)cccc1

# Alphas + some NCAAs
# "[C*]C(=O)N", "[C*]C(=O)[O-]", "[C*]CC(=O)[O-]", "[C*]CC(=O)N", "[C*]CCC[N+]", "[C*]CC[N+]", "[C*]CCNC(=[N+])N", "[C*]C(C)C",


# phenylalanine based stubs
c_repl[Nof(c_repl)+1] = Collection( "what" "[c;D2]1[c;D2][c;D2][c;D2][c;D2][c;D3]1[$(CC(C=O)[N;D2])]" \
                                	"with" { "[C*]c1ccc(O)cc1", "[C*]c1ccc(I)cc1", "[C*]c1c(Cl)cccc1", "[C*]c1cc(Cl)ccc1", "[C*]c1ccc(Cl)cc1", "[C*]c1c(F)c(F)c(F)c(F)c1(F)", "[C*]c1ccc(C#N)cc1", "[C*]c1ccc(C)cc1", "[C*]c1c(C)cccc1", "[C*]c1cc(C)ccc1", "[C*]c1c(C)cc(C)cc1", "[C*]c1ccc(C(C)(C)C)cc1", "[C*]c1cc(OC)ccc1", "[C*]c1ccc(OC)cc1", "[C*]c1cc(F)cc(F)c1", "[C*]c1c(C(F)(F)F)cccc1", "[C*]c1cc(C(F)(F)F)ccc1", "[C*]c1ccc(C(F)(F)F)cc1", "[C*]c1c[nH]c2ccccc21", "[C*]c1c[nH]c2ncccc21"} )

# N_methyl phenylalanine based stubs
c_repl[Nof(c_repl)+1] = Collection( "what" "[c;D2]1[c;D2][c;D2][c;D2][c;D2][c;D3]1[$(CC(C=O)[N;D3])]" \
                                	"with" { "[C*]c1ccc(O)cc1", "[C*]c1ccc(I)cc1", "[C*]c1c(Cl)cccc1", "[C*]c1cc(Cl)ccc1", "[C*]c1ccc(Cl)cc1", "[C*]c1c(F)c(F)c(F)c(F)c1(F)", "[C*]c1ccc(C#N)cc1", "[C*]c1ccc(C)cc1", "[C*]c1c(C)cccc1", "[C*]c1cc(C)ccc1", "[C*]c1c(C)cc(C)cc1", "[C*]c1ccc(C(C)(C)C)cc1", "[C*]c1cc(OC)ccc1", "[C*]c1ccc(OC)cc1", "[C*]c1cc(F)cc(F)c1", "[C*]c1c(C(F)(F)F)cccc1", "[C*]c1cc(C(F)(F)F)ccc1", "[C*]c1ccc(C(F)(F)F)cc1", "[C*]c1c[nH]c2ccccc21", "[C*]c1c[nH]c2ncccc21"} )


# N_methyl Aminobutyiuc acid based stubs
c_repl[Nof(c_repl)+1] = Collection( "what" "[C;D1][$([C;D2]C(C=O)[N;D3])]" \
                                	"with" { "[C*]C(=O)N", "[C*]C(=O)[O-]", "[C*]CC(=O)[O-]", "[C*]CC(=O)N", "[C*]CCC[N+]", "[C*]CC[N+]", "[C*]CCNC(=[N+])N", "[C*]CC", "[C*]CCC", "[C*]C=C", "[C*]C#C", "[C*]C1CC1", "[C*]C1CCC1", "[C*]C1CCCC1", "[C*]C1CCCCC1", "[C*]C(C)(C)C", "[C*]Cc1ccccc1", "[C*]C(C)C"} )

# Aminobutyiuc acid based stubs
c_repl[Nof(c_repl)+1] = Collection( "what" "[C;D1][$([C;D2]C(C=O)[N;D2])]" \
                                	"with" { "[C*]C(=O)N", "[C*]C(=O)[O-]", "[C*]CC(=O)[O-]", "[C*]CC(=O)N", "[C*]CCC[N+]", "[C*]CC[N+]", "[C*]CCNC(=[N+])N", "[C*]CC", "[C*]CCC", "[C*]C=C", "[C*]C#C", "[C*]C1CC1", "[C*]C1CCC1", "[C*]C1CCCC1", "[C*]C1CCCCC1", "[C*]C(C)(C)C", "[C*]Cc1ccccc1", "[C*]C(C)C"} )


# N Methyl Valine based stubs
###### Need to check the chirality of isoleucine, first element in array
c_repl[Nof(c_repl)+1] = Collection( "what" "[$([C;D3]C(C=O)[N;D3])]([C;D1])([C;D1])" \
                                	"with" { "[C*](C)CC", "[C*](C)(C)(C)", "[C*]1CC1", "[C*](CC)CC"} )

#  Valine based stubs
###### Need to check the chirality of isoleucine, first element in array
c_repl[Nof(c_repl)+1] = Collection( "what" "[$([C;D3]C(C=O)[N;D2])]([C;D1])([C;D1])" \
                                	"with" { "[C*](C)CC", "[C*](C)(C)(C)", "[C*]1CC1", "[C*](CC)CC"} )

# Oxazole based stubs
c_repl[Nof(c_repl)+1] = Collection( "what" "[$([C;D1]C([N;D2])c1occ(C(=O))n1)]" \
                                	"with" { "[C*]C(=O)N", "[C*]C(=O)[O-]", "[C*]CC(=O)[O-]", "[C*]CC(=O)N", "[C*]CCC[N+]", "[C*]CC[N+]", "[C*]CCNC(=[N+])N", "[C*]C(C)C", "[C*](C)CC", "[C*]c1ccc(O)cc1", "[C*]c1ccccc1", "[C*](C)C", "[C*]c1c[nH]c2ccccc21", "[C*]CSC"} )

# thiazole based stubs
c_repl[Nof(c_repl)+1] = Collection( "what" "[$([C;D1]C([N;D2])c1scc(C(=O))n1)]" \
                                	"with" { "[C*]C(=O)N", "[C*]C(=O)[O-]", "[C*]CC(=O)[O-]", "[C*]CC(=O)N", "[C*]CCC[N+]", "[C*]CC[N+]", "[C*]CCNC(=[N+])N", "[C*]C(C)C", "[C*](C)CC", "[C*]c1ccc(O)cc1", "[C*]c1ccccc1", "[C*](C)C", "[C*]c1c[nH]c2ccccc21", "[C*]CSC"} )


# Sarcosine based Stubs
c_repl[Nof(c_repl)+1] = Collection( "what" "[$([C;D1]N[C;D2][C;D3](=O))]" \
                                	"with" { "[C*]CC1=C(Cl)C=C(Cl)C=C1", "[C*]CC1=CC(Cl)=CC=C1", "[C*]CC1=CC=CC=C1", "[C*]CC(F)(F)F", "[C*]C(F)(F)F", "[C*]C#N", "[C*]CCC(F)(F)F", "[C*]C1COC1", "[C*]CC1=C(F)C=C(Br)C=C1", "[C*]CC1=C(F)C=CC=C1", "[C*]CC1=CC(F)=CC=C1", "[C*]C1=CC=C(C2=CC=CC=C2)C=C1", "[C*]C1=C(Cl)C=CC(F)=C1", "[C*]C1=CC(C2=CC=CC=C2)=CS1", "[C*]C1=C(F)C(F)=C(F)C(F)=C1(F)", "[C*]C1=CC(C2=CC=CC=C2)=CC=C1", "[C*]C1=C(F)C=CC=C1", "[C*]C1=C(F)C=CC=C1(F)", "[C*]C1=C(F)C=CC=C1", "[C*]C1=CC(F)=C(F)C(F)=C1", "[C*]C1=C(Br)C=CC(OC)=C1", "[C*]C1=C(F)C(F)=CC=C1", "[C*]CCC", "[C*]C1=CC(F)=C(Cl)C=C1", "[C*]C1=CC(Cl)=C(F)C=C1", "[C*]C1=CC(Cl)=CC(F)=C1", "[C*]CC1=CC=C(F)C=C1", "[C*]CC1=CC(OC)=CC=C1", "[C*]C1=C(O)C=CC(Cl)=C1", "[C*]C(C)C", "[C*]CC1=CC=C(Br)C=C1", "[C*]CC1=CC=C(OC)C=C1", "[C*]C1=C(F)C(Cl)=CC=C1", "[C*]CC1=CC=C(Cl)C=C1", "[C*]CC1=C(OC)C=CC(OC)=C1", "[C*]CC1=C(OC)C=CC=C1", "[C*]CC1=CN=C(C)C=C1", "[C*]CC1=C(Br)C=CC=C1", "[C*]COC", "[C*]CCOC", "[C*]C(C)(C)OC", "[C*]C1=CC=C(S(=O)(=O)C)C=C1", "[C*]C1=CC(Cl)=CC=C1", "[C*]C1=C(C)C=CC=C1", "[C*]C1=CC=CC=C1", "[C*]C1=C(Cl)C=CC=C1", "[C*]C1=C(O)C=CC(Br)=C1", "[C*]CC1=CC(C(F)(F)F)=CC=C1", "[C*]COC1=C(C)C=CC=C1(C)", "[C*]CC1=CC=C(C(F)(F)F)C=C1", "[C*]CC1=CC=C(C)C=C1", "[C*]CC1=C(F)C=C(F)C=C1", "[C*]CC1=C(F)C=CC(Cl)=C1", "[C*]CC1=C(F)C=C(Cl)C=C1", "[C*]CC1=C(F)C=CC=C1(F)", "[C*]C1CCOCC1", "[C*]C1=CC=C(S(=O)(=O)N2CCOCC2)C=C1", "[C*]1CCS(=O)(=O)CC1", "[C*]C1=CC=C(S(=O)(=O)N2CCCC2)C=C1", "[C*]C1=C(C2=CC=CC=C2)C=CC=C1", "[C*]C#C", "[C*]C1=CC=C([N+](=O)[O-])C=C1", "[C*]C1=C(C)C=C(Cl)C=C1", "[C*]CC", "[C*]C1=CC(Br)=CC=C1", "[C*]C1=C(Cl)C(Cl)=CC=C1", "[C*]C1=CC=C(Br)C=C1", "[C*]COCC", "[C*]C1=C(F)C(OC)=C(F)C=C1", "[C*]C1=CC=C(I)C=C1", "[C*]C1=CC=C(C)C(Cl)=C1", "[C*]C1=C(Cl)C=CC(Cl)=C1", "[C*]C1=CC=C(OC2=C(Cl)C=C(Cl)C=C2)C=C1", "[C*]C1=CC=CC(I)=C1", "[C*]C1=CC(OC2=CC(Cl)=CC(Cl)=C2)=CC=C1", "[C*]C1=CC=NC(OC)=C1", "[C*]C1=CC=C(OC)N=C1", "[C*]C1=CC=C(Cl)C=C1", "[C*]C1=NC(C(C)(C))=CS1", "[C*]C1=CC=C(C(F)(F)F)C=C1", "[C*]C1=CC(Cl)=C(OC)C=C1", "[C*]C1=C(Cl)C=CC(Br)=C1", "[C*]C1=C(C)SC(C)=C1", "[C*]C1=CC=CO1", "[C*]C1=COC=C1", "[C*]C1=C(Br)C=C(F)C=C1", "[C*]C1=CC(C)=C(Br)C=C1", "[C*]C1=CC(Br)=C(Cl)C=C1", "[C*]C1=CC=C(O)C(Br)=C1", "[C*]C1=C(F)C=C(F)C=C1", "[C*]C1=C(Br)C=CC=C1", "[C*]C1=CC(Br)=C(OC)C=C1", "[C*]C1=CC=CS1", "[C*]C1=C(F)C(F)=C(F)C=C1", "[C*]C1=C(F)C(OC)=C(F)C(F)=C1", "[C*]C1=CC(OC)=CC(OC)=C1", "[C*]C1=CC=C(OC)C=C1", "[C*]C1=CC(OC)=C(OC)C=C1", "[C*]C1=CC(OC)=C(OC)C(OC)=C1", "[C*]C1=CC=C(S(=O)(=O)N)C=C1", "[C*]C1CCN(C(=O)C(C)C)CC1", "[C*]CC1=CC(F)=CC(F)=C1", "[C*]CC1=C(F)C=CC(F)=C1",  } )
                                	# can be extended further with other replacements

# Beta_phenylalanine based stubs
c_repl[Nof(c_repl)+1] = Collection( "what" "[c;D2]1[c;D2][c;D2][c;D2][c;D2][c;D3]1[$(CC(CC=O)[N;D2])]" \
                                	"with" { "[C*]c1ccc(Cl)cc1", "[C*]c1ccc(F)cc1", "[C*]c1ccc(I)cc1", "[C*]c1ccc(Br)cc1", "[C*]c1cc(Cl)ccc1", "[C*]c1cc(C)ccc1", "[C*]c1c(Cl)cccc1", "[C*]c1cc(Cl)c(Cl)cc1", "[C*]c1cc(F)c(F)cc1(F)", "[C*]c1c(F)c(F)c(F)c(F)c1(F)" } )

n_out = 0

for ichem=1,Nof(ix)
	# read next entry in 3D
	read mol table ix[ichem] name="chem"
	read mol chem.mol[1] name="pep" delete
	#print "Processing: " ichem " out of " nofComp
	# convert to ICM
	build hydrogen
	set type mmff
	convert auto
	delete chem_out   # results for this row

	### Loop over the number of things in the c_repl array
	for i=1,Nof(c_repl)

    	s_what = c_repl[i,"what"]
    	S_with = c_repl[i,"with"]

    	#print s_what S_with Nof(c_repl) Nof(S_with)

    	find chemical all s_what a_   # search for all pattern
    	as_match = as_out

    	#print s_what, "number_of_matches=" Nof(as_match)

    	if (Nof(as_match) > 0) then

        	#print s_what

        	n_match = Nof( Chemical( as_match exact ) molecule )[1]	# number of matches 1,2,3...

        	##### Call the function here to get all combinations...
        	all_comb = getAllComb( n_match Nof(S_with) )

        	#print "n_match=" n_match, "n_comb=" n_comb, "n_comb_full=" n_comb_full
        	#print all_comb, Nof(all_comb)

        	#### Loop over all combinations...
        	for j=1,Nof(all_comb)

            	#print all_comb[j]

            	copy a_ "tmpcopy" delete selection
            	set object a_tmpcopy.


            	## label locations that need to be subsitituted
            	for pos=1,n_match
                	if ( all_comb[j][pos] != 0 ) then
                    	set field Select( a_// & as_match molecule pos ) name="subst_" + pos
                	endif
            	endfor

            	## make the subsitutsion
            	for pos=1,n_match
                	if ( all_comb[j][pos] != 0 ) then
                    	# get selection
                    	S_WITH_IDX = all_comb[j][pos]
                    	as_graph = Select( a_// "subst_" + pos)
                    	s_selfGroup = ""

                    	# remove selection and prepare for subsitution
                    	e3dPrepareGroupReplace as_graph s_selfGroup
                    	as3_out = a_//

                    	# attach new group
                    	#show S_with[S_WITH_IDX]
                    	modifyGroupSmiles as_graph S_with[S_WITH_IDX] yes yes no
                    	vs_out = v_//!vt* & as_graph

                    	# optmize new group variables
                    	if (Nof(vs_out) > 0) then
                        	ssearch  mute vs_out "vw,14,to,hb,ts" selftether=as3_out | a_//!vt* [1]
                    	endif

                    	nsubst += 1
                	endif
            	endfor
            	if (nsubst > 0) then
                	add chem_out chem   # copy original SDF fields
                	chem_out.mol[ Nof(chem_out) ] = Chemical( a_ exact hydrogen )
            	endif
            	delete a_tmpcopy.
        	endfor
    	endif
	endfor
	delete a_

	if (Nof(chem_out)) then
    	write table mol append chem_out s_outfile
    	n_out += Nof(chem_out)
	endif
endfor

printf " Info> %d molecules stored in %s\n", n_out, s_outfile

quit
```

At this point in the process, each sequence in the library contains one dichlorobenzene stub sidechain and myriad ncAA mutations at the remaining positions.

*Virtual Screening*: We developed a two-step virtual screening approach tailored to the designed macrocycles described herein. In the first step, we generate a candidate macrocycle : MCL1 pose in a custom grid-based packing script for each designed macrocycle in the library. In the second step, we compute and compare the docking scores of a subset of designed macrocycles in Rosetta. Ultimately we developed this approach to reduce the number of designed poses needed to be evaluated in Rosetta, which requires a laborious parametrization of each ligand and (comparably) expensive minimization of the designed macrocycle.

*Grid-based packing*: We formulated our ligand docking search as an inverse packing problem. For each macrocycle, we first align the macrocycle’s dichlorobenzene stub to the X-ray crystallographic structure of a known ligand that contains a dichlorobenzene bound to MCL1 (PDBID: 5MEV). We then sample torsion angles for each rotatable bond in side chains attached to the macrocycle, keeping the cartesian coordinates of the stub fixed in place. For each sampled conformer, we evaluate the interaction energy using a grid-based lookup into a pre-computed grid that stores leonard jones, electrostatic, and solvation energies on a grid of user specified resolution (typically 0.25 Å).

The below command is used to generate the pre-computed spherical grid:

python make_grid.py \

--grid_rad 15.0 \

--grid_resl 0.25 \

--grid_origin "90 29 55" \

--target </path/to/target.pdb> \

-o </path/to/output/>

where --grid_origin is a string of “x y z” coordinates to center the sphere of --grid_rad angstroms and --target is the full path to the protein to prepare the grid against.

The below command is used then used to perform the packing search of a macrocycle in the grid:

python vector_dock_align.py \

--input DICL_inputs_0.sdf \

--output </path/to/outputs/> \

--stub </path/to/stub.sdf> \

--target </path/to/target.pdb> \

--smiles '[#17][#6]1[#6]([#17])[#6]([#1])[#6]([#6])[#6]([#1])[#6]1[#1]’ \

--align '[#17][#6]1[#6]([#17])[#6]([#1])[#6]([#6])[#6]([#1])[#6]1[#1]' \

--grid <path/to/precomputed.grid>

where --stub is the full path to an sdf file of the dichlorobenzene extracted from from the ligand within PDBID 5MEV; --target is the full path to the protein target only; --smiles and --align are smarts/smiles pattern used to identity the atoms within the docking ligand and stub that belong to the dichlorobenzene stub; and --grid is the full path to the precomputed grid prepared in the previous step.

*Rosetta Energy Calculations:* We prioritized designs for evaluation in Rosetta based on their grid scores from the previous step. We selected the top 1% of designs by grid score (250,000 designs form the large library, 25,000 designs from the small library), parameterized each ligand in Rosetta’s generalized potential, and evaluated each ligand's Docking score in a custom pyrosetta script. This custom script first minimizes the binding pose identified from the grid-based search into Rosetta's energy function, then calculates the dG of binding using the GALigandDock mover. The below command shows how to evaluate each ligand:

python gald_eval.py \

--params_file </path/to/lig.params> \

--target_file </path/to/target.pdb> \

--output_dir </path/to/output_dir/>

We then ranked each design by their Rosetta calculated dG and visually inspected approximately 1000 of the top scoring designs from each library.

**Figure S1.** All 130 chemicals for which potential energy surfaces were calculated. The chemotype bin for each chemical is listed next to its abbreviated name. See table S14 for SMILES strings of the depicted monomers.

**Figure S2**. Chemotype distribution of unique sequences sampled across all 4-residue macrocycles discovered in our expansive search (left), all 3- and 4-residue macrocycles in the Cambridge Structural Database (middle), and all 3- and 4- residue macrocycles in the PubChem database (right).

**Figure S3**. Many conformers of many sequences within a chemotype disproportionately sample distinct backbone torsion bins. (A) Cartoon of the torsion binning scheme. Each conformer of each sequence belonging to a given chemotype (aaa in this example) is built and its backbone torsion angles are measured and binned on 60 degree intervals. The number of conformers that the 16 most densely sampled bins are displayed in the resulting bar chart. (B) The 16 most sampled bins for several 3-residue and 4-residue macrocycles. The non-uniform sampling is apparent.

**Figure S4.** Approximately one half of the unique macrocycles enumerated here obey the rule of five. (**A**) Distribution of unique sequences obeying (Ro5) and disobeying (bRo5) Lipinski's rule of 5. (**B**) Distribution of macrocycles molecular weight and calculated atomic log(P). (**C**) Histogram showing the distribution of hydrogen bond acceptor atoms in each macrocycle. (**D**) Histogram showing the distribution of hydrogen bond donor atoms in each macrocycle.

**Figure S5.** Impact of sidechain substitution on molecular shape. (**A**) Principal moment of inertia analysis of every atom in the lowest energy conformer for a handful of sequences across three chemotypes. Distinct preferences for PMI ratios are apparent within each chemotype. (**B**) Superposition of the lowest energy conformers of the macrocycles aaap-PRO-PHE_pp-mTIC-AMACBEN3 (green) and aaap-PRO-mPHE_pp-mTIC-AMACBEN3 (cyan). These diastereomers orient their phenylalanine and Tic monomers in a similar arrangement despite the differing alpha stereocenters within their respective phenylalanine monomers. This results in macrocycles that have very similar shapes despite the epimerization and distinct torsional bins they populate.

**Figure S6.** The low-energy conformer identified from the hash-generated ensembles are very similar to the X-ray crystallographic structure of the macrocycle. The backbone atoms are highlighted per Figure 1A in the main text. In each of the energy landscapes, RMSD is calculated to the lowest energy conformer sampled in the ensemble. Each blue dot represents the endpoint of an AIMNet minimization of a single conformer generated by the hash-based assembly. The predicted low-energy conformers for each macrocycle are depicted as green sticks. Crystallographic coordinates for each macrocycle are depicted as grey sticks. For cyclotrisarcosine, the X-ray crystallographic structure contains two distinct conformers of the macrocycle in the asymmetric unit. These conformers are related to one-another through a mirror inversion. These two conformers are sampled in the hash-generated ensemble and are nearly identical in energy. These two predicted conformers are depicted in green and blue and are superimposed on the asymmetric unit of cyclotrisarcosine.

**Figure S7.** Energy landscapes of macrocycles whose unminimized ensembles sample few torsion bins frequently contain deep energy minima that sample a single state with Pnear > 0.8. Ensembles of 3 residue macrocycles were minimized (1735 sequences from macrocycles that sampled < 5 bins, 889 from macrocycles that sampled > 100 bins) and the quality of the resulting landscapes were assessed using Pnear. Energy landscapes of macrocycles whose unminimized ensembles sample many torsion bins often contain shallow energy minima that sample multiple states.

**Figure S8.** The NMR ensemble or X-ray crystallographic structures of three designed macrocycles are not close to their predicted low-energy conformation. Design models are colored green. Experimental coordinates are colored grey. Design models are depicted in green sticks. The 20 lowest-energy conformers from unconstrained MD simulations are depicted as lines. X-ray crystallographic structures are depicted as sticks. The distance-constrained ensemble of aaag **1** contains three distinct conformers that all differ from the predicted low energy conformer of the molecule. The distance-constrained ensemble of aabs **1** contains a single conformer, at the level of backbone torsion angles, that is stabilized by a hydrogen bond between the carbonyl of the aminobutyric acid monomer and the NH of the cysteine-containing monomer.

Table S1.

**Figure S9.** X-ray and NMR structures of two locally encoded macrocycles are both very close to their respective design model. Column I: chemical structure colored by chemotype; Column II: design model; Column III: superposition of the design model and the NMR ensemble models; Column IV: Superposition of the design model and the X-ray crystallographic structures. Design models are depicted in green, experimental coordinates are depicted in grey.

**Figure S10.** *Cis*-amides are a recurrent motif amongst 4-residue macrocycles constructed of primarily alpha amino acids. (**A**) Fraction of conformers across each sequence within the specified chemotype containing only trans amides (orange) or at least 1 *cis*-amide (blue). *cis*-Amides are present in many of the conformers of macrocycles sampled amongst chemotypes comprised of three alpha-amino acids and one unnatural amino acid, especially for chemotypes constructed from backbones that contain many fixed torsion angles (e.g. 3-aminobenzoic acid / f containing macrocycles or 4-aminomethylphenylacetic acid / q containing macrocycles). Long unconstrained backbones, such as epsilon-amino acids (e), and backbones with obvious turn-inducing motifs, such as 2-aminobenzoic acids (c) and 1,2- or 1,3- aminomethylphenylacetic acids (m, p respectively), alleviate the strain associated with cis amides, which primarily involve tertiary amides. (**B**) Ramachandran plot of every alpha amino acids with a *cis*-amide from chemotypes in panel A. Green points represent *N*-alkylated amino acids (e.g. *N*-methylalanine), orange points represent proline-like amino acids, and blue points represent *N*-proto amino acids (e.g. alanine). These tertiary amides populate 4 distinct regions of ramachandran space, regardless if they are from proline-like residues, *N*-methylated residues, or peptoids; the same regions of ramachandran space are also sampled by *N*-proto amino acids that contain *cis*-amides. Representative conformers of D-proline from within two of the four discrete bins are depicted as sticks. These conformers promote the rapid turning of the backbone amongst these macrocycles. These conformers are not privileged to proline-like amino acids, but are instead sampled by most alpha amino acids within these macrocycles. These specific combinations of phi and psi torsion angles, in conjunction with the *cis*-amide, yield conformers of alpha amino acids that promote rapid turning of the backbone within a single residue; the same regions of ramachandran space, but with *trans*-amides, produce much more extended conformers of alpha amino acids.

**Figure S11.** aaam **6** mimics the spacing and orientation of residues at i,i+4 of alpha-helices. X-ray crystallographic structure of aaam **6** (left) superimposed on an idealized alpha-helix (right).

**Figure S12.** PAMPA results for all prepared macrocycles. The dashed red line is placed at P_app_ = 1 x 10^-6^ cm/s. Compounds in red were not detected in the acceptor well via LCMS after *ca.* 19 hours of incubation. Data are mean +/- S.D.

Figure **S13**. Process flow diagram for Boltzmann Adaptive Sampling protocol for monomeric residues. The process iterates indefinitely until sufficient sampling is achieved which is checked periodically. Heuristically, we found that 10*10^N^ samples is sufficient where N is the total number of degrees of freedom. The number of centers sampled per iteration (Y) should be dynamically controlled to balance the time calculating the Delaunay triangulation and evaluating the energy of molecules. The loss function is the Volume Adjust Boltzmann Weighted Probability which is calculated by multiplying the volume of a simplex by the mean of the unnormalized Boltzmann probability for each vertex of the simplex.

Figure **S14**. Ramachandran plot of alanine with N-terminal acetylation and C-terminal methyl amidation generated with Boltzmann Adaptive Sampling. Each vertex of the Delaunay Triangulation is a sampled point. The surface contour is generated with a linear interpolation of the resulting Delaunay Triangulation. The potential surface is colored by relative energy (kcal/mol) to the lowest energy conformation sampled on the surface.

 Figure **S15**. A plot of Kullback-Libler (KL) Divergence versus sample count for 10 trajectories of conformer ensemble sampling with (blue) and without (red) adaptive sample for glycine (left), alanine (center), and alpha-aminoisobutyric acid (right). The divergence of the ensemble is calculated between checkpoints during sampling and the final exhaustive ensemble. The potential energy surfaces for each residue are shown as an insert within each respective plot. The BAS-guided sampling converges in fewer sample points than a uniform grid-based sampling scheme.

 Figure **S16**. Impact of hashtable resolution on quality of macrocyclic closures in two residue macrocycles. Hashtables for each monomer were constructed at high (0.5 Å, 10 degree) medium (0.75 Å, 12.5 degree), low (1.0 Å, 15 degree) and very low (2.0 Å, 30 degree) cartesian and origin resolutions. The error in NCO alignment in each of the resulting conformers were calculated and plotted as KDE normalized histograms. The higher the resolution of the hashtables, the lower the RMSD error in fragment alignment.

Figure **S17**. Identification of candidate low energy sequences for each torsion bin sampled by base residues in a chemotype. (**A**) Diagram of algorithm to identify potential low energy sequences. (**B**) Example of process used to identify lowest energy rotamer at each monomer within a single torsion bin representative of the aaas-SAR_pp-SAR-AGLY-SUGA macrocycle. First the backbone torsion angles of the bin representative are calculated. Next, at each monomer, the lowest energy rotamer is identified for each substitution possible at the monomer, given the backbone torsion angles in the torsion bin representative. Finally, the candidate sequence is constructed by identifying the substitution with the lowest energy at each monomer. The monomers in the candidate sequence are colored to match the torsion angles displayed on the torsion bin cluster.

Figure **S18**. Residual of the linear regression model fit to predict AIMNet energies from sequence composition (top). Z Score analysis for all 8,280 macrocycles used to construct the linear regression model (bottom). Macrocycles whose Pnear > 0.8, and Zscore <-2.0 were manually inspected and a subset were chemically synthesized.

Figure **S19.** *De novo* designed macrocycles are largely stable to serum proteases compared to linear peptide hormones. (**A**) Human serum stability of *de novo* designed small macrocycles. The indicated peptides were incubated with reconstituted human serum for 24h and the % of peptide remaining of each was measured using LC-MS after the indicated lengths of time. Data are mean +/- S.D. (**B**) Violin plot showing the distribution of percent remaining for the small macrocycles (denoted minicycle) and the peptide hormones. Each rug represents each individual replicate.

Figure **S20.** Biochemical inhibition of members of the histone deacetylase enzyme family by de novo designed small macrocycles. Inhibition of (A) HDAC1, (B) HDAC2, (C) HDAC3, (D), HDAC4, (E) HDAC5, (F) HDAC6, (G) HDAC7, (H) HDAC8, (I) HDAC8, and (J) HDAC11. The 10-dose singlet titrations for the designed macrocycles are plotted in the first two columns. The 10-dose singlet titration of the positive control is plotted in the third column. Curve fits were performed where the enzyme activities at the highest concentration of compounds tested were less than 65%. N.D. indicates that no inhibitory activity was observed for the specified set of designed macrocycles. Fitted IC50s are tabulated in table S11 below.

Figure **S21**. Biochemical inhibition of SARS-CoV-2 main protease by *de novo* designed small macrocycles. 10-Dose singlet titrations for the designed macrocycles are plotted and fit where the enzyme activities at the highest concentration of compounds tested were less than 65%. Fitted IC50s are tabulated in table S12 below.

Figure **S22**. Biochemical inhibition of Mcl-1 : Bak interface by *de novo* designed small macrocycles from the large (A) library and the small (B) library. 10-Dose singlet titrations for the designed macrocycles are plotted and fit where the fraction of bound labeled peptide at the highest concentration of compound tested were less than 65%. Fitted IC50s are tabulated in table S13 below.

Figure **S23**. Distribution of a random 25k designed macrocycles’ Rosetta docking scores from both the large and small virtual libraries. The larger library produced better scoring designs than the small library. Table S13 summarizes the experimental results of the virtual screens.

Figure **S24**. Histogram of purity of synthesized compounds as determined by integration of peaks in HPLC chromatograms.

**Supplementary Table 1.** ROESY-based distance constraints for aaaq 1

| residue 1 | atom 1 | residue 2 | atom 2 | calculated NOE (Å) | NOE lower bound (Å) | NOE upper bound (Å) |
| --- | --- | --- | --- | --- | --- | --- |
| AIB | NH | AMACBEN4 | NH | 2.999 | 2.7 | 3.3 |
| D-ASN | NH | FPA | NH | 2.83 | 2.55 | 3.11 |
| D-ASN | HA | FPA | NH | 2.637 | 2.37 | 2.9 |
| AIB | NH | FPA | HA | 2.378 | 2.14 | 2.62 |
| AIB | NH | FPA | HB1 | 3.165 | 2.85 | 3.76 |
| AIB | NH | FPA | HB2 | 3.418 | 2.85 | 3.76 |

**Supplementary Table 2.** ROESY-based distance constraints for aaap 3

| residue 1 | atom 1 | residue 2 | atom 2 | calculated NOE (Å) | NOE lower bound (Å) | NOE upper bound (Å) |
| --- | --- | --- | --- | --- | --- | --- |
| AMACBEN3 | NH | AMACBEN3 | HArr_1 | 1.855 | 1.67 | 2.04 |
| AMACBEN3 | NH | D-NMPhe | HA | 2.024 | 1.82 | 2.23 |
| D-NMPhe | HA | DPro | HA | 1.48 | 1.33 | 1.63 |
| PPS | HA | DPro | HD1 | 2.197 | 1.98 | 2.24 |
| PPS | HA | DPro | HD2 | 2.426 | 2.18 | 2.67 |

**Supplementary Table 3.** ROESY-based distance constraints for aabc 1

| residue 1 | atom 1 | residue 2 | atom 2 | calculated NOE (Å) | NOE lower bound (Å) | NOE upper bound (Å) |
| --- | --- | --- | --- | --- | --- | --- |
| PHE | NH | BEN2 | NH | 3.469 | 3.1 | 3.8 |
| BPRO | HMs | BEN2 | NH | 2.677 | 2.4 | 2.9 |
| PHE | NH | BPRO | HD1 | 3.671 | 3.3 | 4 |
| PHE | NH | BPRO | HD2 | 3.671 | 3.3 | 4 |
| PHE | NH | AZE | HA | 2.549 | 2.3 | 2.8 |
| PHE | HA | BPRO | HD1 | 1.323 | 1.2 | 1.5 |
| PHE | HA | BPRO | HD2 | 1.323 | 1.2 | 1.4 |
| AZE | HG1 | BEN2 | HArr4 | 2.186 | 2 | 2.4 |

**Supplementary Table 4.** ROESY-based distance constraints for aaap 4

| residue 1 | atom 1 | residue 2 | atom 2 | calculated NOE (Å) | NOE lower bound (Å) | NOE upper bound (Å) |
| --- | --- | --- | --- | --- | --- | --- |
| TIC | HA | D-NMVAL | HA | 1.782 | 1.56 | 1.9 |
| PIP | HA | TIC | HE1 | 1.912 | 1.72 | 2.1 |
| AMACBEN3 | HB1 | PIP | HE1 | 1.945 | 1.75 | 2.14 |
| AMACBEN3 | NH | D-NMVAL | HA | 2.272 | 2.04 | 2.5 |
| AMACBEN3 | NH | AMACBEN3 | HArr_1 | 2.179 | 1.96 | 2.4 |
| PIP | HE2 | AMACBEN3 | HArr_1 | 2.993 | 2.69 | 3.29 |

**Supplementary Table 5.** ROESY-based distance constraints for aaam 2

| residue 1 | atom 1 | residue 2 | atom 2 | calculated NOE (Å) | NOE lower bound (Å) | NOE upper bound (Å) |
| --- | --- | --- | --- | --- | --- | --- |
| AMACBEN2 | NH | TBG | NH | 4.224 | 3.8 | 4.65 |
| AMACBEN2 | NH | TBG | HA | 2.315 | 2.08 | 2.55 |
| TBG | NH | HPR | HA | 3.55 | 3.2 | 3.91 |
| VAL | HA | HPR | HA | 1.979 | 1.78 | 2.18 |
| HPR | HA | VAL | HB | 3.582 | 3.22 | 3.94 |

**Supplementary Table 6.** ROESY-based distance constraints for aalm 1

| residue 1 | atom 1 | residue 2 | atom 2 | calculated NOE (Å) | NOE lower bound (Å) | NOE upper bound (Å) |
| --- | --- | --- | --- | --- | --- | --- |
| NVL | NH | AMACBEN2 | HB1 | 2.567 | 2.31 | 2.82 |
| NVL | NH | AMACBEN2 | HB2 | 3.411 | 3.07 | 3.75 |
| ACBEN3 | NH | TIC | HA | 3.045 | 2.74 | 3.35 |
| ACBEN3 | NH | TIC | HE1 | 2.778 | 2.5 | 3.06 |
| ACBEN3 | NH | ACBEN3 | HArr_1 | 2.177 | 1.96 | 2.39 |
| TIC | HE2 | TIC | HB2 | 3.138 | 2.82 | 3.44 |
| AMACBEN2 | NH | ACBEN3 | HB1 | 2.367 | 2.13 | 2.6 |
| AMACBEN2 | NH | ACBEN3 | HB2 | 3.878 | 3.49 | 4.27 |
| ACBEN3 | HArrs_3 | ACBEN3 | HB1 | 3.395 | 3.06 | 3.73 |
| ACBEN3 | HArrs_3 | ACBEN3 | HB2 | 2.503 | 2.25 | 2.75 |

**Supplementary Table 7.** ROESY-based distance constraints for aabc 2

| residue 1 | atom 1 | residue 2 | atom 2 | calculated NOE (Å) | NOE lower bound (Å) | NOE upper bound (Å) |
| --- | --- | --- | --- | --- | --- | --- |
| BEN2 | NH | D-TBA | NH | 3.886 | 3.5 | 4.27 |
| BEN2 | NH | LEU | NH | 3.726 | 3.35 | 4.1 |
| LEU | NH | D-TBA | NH | 3.362 | 3.03 | 3.7 |
| BEN2 | NH | BEN2 | HArr1 | 3.059 | 2.75 | 3.36 |
| BEN2 | NH | B3VAL | NH | 3.016 | 2.71 | 3.32 |
| D-TBA | NH | BEN2 | HArr4 | 2.257 | 2.03 | 2.48 |
| LEU | NH | B3VAL | NH | 2.311 | 2.08 | 2.54 |
| LEU | NH | D-TBA | HA | 2.432 | 2.19 | 2.68 |
| B3VAL | NH | LEU | HA | 2.973 | 2.68 | 3.27 |
| BEN2 | NH | B3VAL | HB1 | 3.17 | 2.85 | 3.49 |
| BEN2 | NH | B3VAL | HB2 | 3.431 | 3.09 | 3.77 |

**Supplementary Table 8.** ROESY-based distance constraints for aaam 1

| residue 1 | atom 1 | residue 2 | atom 2 | calculated NOE (Å) | NOE lower bound (Å) | NOE upper bound (Å) |
| --- | --- | --- | --- | --- | --- | --- |
| AMACBEN2 | NH | AMACBEN2 | Harr1 | 3.453 | 3.11 | 3.8 |
| AMACBEN2 | NH | tBuGly | NH | 3.976 | 3.58 | 4.37 |
| AMACBEN2 | Harr3 | AMACBEN2 | Harr4 | 2.442 | 2.2 | 2.69 |
| AMACBEN2 | NH | tBuGly | HA | 2.35 | 2.12 | 2.59 |
| AMACBEN2 | NH | AMACBEN2 | HA1 | 3.8 | 3.42 | 4.18 |
| AMACBEN2 | NH | AMACBEN2 | HA2 | 3.303 | 2.97 | 3.63 |
| AMACBEN2 | Harr1 | AMACBEN2 | HA1 | 4.066 | 3.66 | 4.47 |
| AMACBEN2 | Harr1 | AMACBEN2 | HA2 | 2.637 | 2.37 | 2.9 |
| tBuGly | NH | LEU | HA | 3.291 | 2.96 | 3.62 |
| tBuGly | NH | FPRO | HA | 3.62 | 3.26 | 3.98 |
| LEU | HA | FPRO | HA | 2.096 | 1.89 | 2.31 |
| AMACBEN2 | HA1 | AMACBEN2 | HB1 | 2.088 | 1.88 | 2.3 |
| LEU | HA | tBuGly | HA | 3.63 | 3.27 | 3.99 |

**Supplementary Table 9.** ROESY-based distance constraints for aaag 1

| residue 1 | atom 1 | residue 2 | atom 2 | calculated NOE (Å) | NOE lower bound (Å) | NOE upper bound (Å) |
| --- | --- | --- | --- | --- | --- | --- |
| D-TIC | HA | D-ALA | HA | 1.895 | 1.71 | 2.08 |
| D-PHE | NH | D-LARD | NH | 3.09 | 2.78 | 3.4 |
| D-ALA | NH | D-TIC | HA | 4.196 | 3.78 | 4.62 |
| D-LARD | NH | D-PHE | HA | 3.468 | 3.12 | 3.81 |
| D-TIC | HA | D-TIC | HB1 | 2.284 | 2.06 | 2.51 |
| D-TIC | HA | D-TIC | HB2 | 2.284 | 2.06 | 2.51 |

**Supplementary Table 10.** ROESY-based distance constraints for aabs 1

| residue 1 | atom 1 | residue 2 | atom 2 | calculated NOE (Å) | NOE lower bound (Å) | NOE upper bound (Å) |
| --- | --- | --- | --- | --- | --- | --- |
| BPIP | HA | ASN | HA | 1.76 | 1.58 | 1.94 |
| ASN | NH | D-ABU | HA | 2.254 | 2.03 | 2.48 |
| SUGA | NH | D-ABU | NH | 3.499 | 3.15 | 3.85 |
| SUGA | NH | BPIP | HA | 2.497 | 2.25 | 2.75 |

**Supplementary Table 11.** Potency of designed HDAC6-selective macrocyclic inhibitors compared to HDAC6-selective inhibitors currently in clinical trials. Each row is a different HDAC, each column, a different inhibitor. Values are IC50s (nM). Values for KA25072, Ricolinostat, and Citairnostat are reproduced from Tsimberidou *et al.*, for ease of comparison [(*52*)](https://paperpile.com/c/4WDLXM/FxCW).

|  | **HDAC** | **macrocycle** | | | | | | | | | | |  | **compound** | | |
| --- | --- | --- | --- | --- | --- | --- | --- | --- | --- | --- | --- | --- | --- | --- | --- | --- |
|  |  | **aaae-**  **HDACi-1** | **aaae-**  **HDACi-2** | **aaap-**  **HDACi-3** | **aahb-**  **HDACi-4** | **aaeb-**  **HDACi-5** | **aapb-**  **HDACi-6** | **aark-**  **HDACi-7** | **aapb-**  **HDACi-8** | **aahb-**  **HDACi-9** | **aahb-**  **HDACi-10** | **aapb-**  **HDACi-11** |  | **KA25072** | **Ricolinostat** | **Citarinostat** |
| **HDAC IC50 (nM)** | **HDAC6** | 3.66 | 1.62 | 1.26 | 789.1 | 2.61 | 40.69 | 7.94 | 5.48 | 39.52 | 70.16 | 10.53 |  | 2.5 | 22 | 2.8 |
|  |  |  |  |  |  |  |  |  |  |  |  |  |  |  |  |  |
|  | **HDAC 1** | 1,150.00 | 815.1 | 842.6 | 4,599.00 | 505.8 | 88.93 | 952.2 | 76.23 | 1,263.00 | 1,424.00 | 68.19 |  | 9895 | 332 | 153 |
|  | **HDAC 2** | 2,903.00 | 1,761.00 | 1,645.00 | 8,525.00 | 1,730.00 | 224.7 | 2,446.00 | 205.9 | 2,559.00 | 3,082.00 | 128 |  | >10000 | 632 | 620 |
|  | **HDAC 3** | 548.5 | 354.4 | 308 | 2,864.00 | 189.3 | 10.22 | 408.8 | 18.25 | 919.9 | 803.7 | 58.66 |  | >10000 | 199 | 268 |
|  | **HDAC 4** | N.D. | N.D. | N.D. | N.D. | N.D. | N.D. | N.D. | N.D. | N.D. | N.D. | N.D. |  | 9613 | >10000 | 9972 |
|  | **HDAC 5** | N.D. | N.D. | 7,744.00 | N.D. | N.D. | N.D. | N.D. | 12,330.00 | N.D. | N.D. | 6,449.00 |  | 1997 | 4310 | 3396 |
|  | **HDAC 7** | N.D. | N.D. | N.D. | N.D. | N.D. | N.D. | N.D. | 24,760.00 | N.D. | N.D. | 15,870.00 |  | 2333 | 5290 | 4031 |
|  | **HDAC 8** | 1,518.00 | 3,401.00 | 697.3 | 2,072.00 | 1,415.00 | 853.9 | 976.4 | 502.7 | 2,720.00 | 2,324.00 | 454.8 |  | 621 | 776 | 155 |
|  | **HDAC 9** | N.D. | N.D. | 14,650.00 | N.D. | N.D. | N.D. | N.D. | N.D. | N.D. | N.D. | N.D. |  | 5648 | >10000 | >10000 |
|  | **HDAC 11** | N.D. | N.D. | 16,830.00 | N.D. | N.D. | N.D. | N.D. | N.D. | N.D. | N.D. | N.D. |  | >10000 | 3433 | 2676 |

**Supplementary Table 12.** Potency of designed macrocyclic inhibitors of SARS-CoV-2 Main Protease. Values are IC50s (M).

| **compound name** | **IC50 (M)** |
| --- | --- |
| aabi-MPRO-1g-linear | >2.00E-04 |
| aabi-MPRO-1j-linear | 1.82E-04 |
| aabi-MPRO-1k-linear | >2.00E-04 |
| aabi-MPRO-1-ga | 1.25E-06 |
| aabi-MPRO-1-ka | 4.07E-06 |
| aabi-MPRO-1-kb | 1.47E-06 |
| aabi-MPRO-1-jb | 1.47E-06 |
| aabi-MPRO-1-gb | 1.22E-06 |
| aabi-MPRO-1-ja | 8.84E-07 |
| aabi-MPRO-1-gc | 1.84E-06 |
| aabi-MPRO-1-kc | 1.27E-06 |
| aabi-MPRO-1-kd | 2.14E-06 |
| aabi-MPRO-1-je | 1.89E-06 |
| aabi-MPRO-1-gd | 2.65E-06 |
| aabi-MPRO-1-jc | 2.37E-06 |
| aabi-MPRO-1-ke | 1.26E-06 |
| aabi-MPRO-1-jd | 1.53E-06 |
| aabi-MPRO-1-g | 3.63E-06 |
| aabi-MPRO-1-k | 1.08E-06 |
| aabi-MPRO-1-j | 1.78E-06 |
| aabi-MPRO-1 | 1.68E-05 |

**Supplementary Table 13.** Potency of designed macrocycle inhibitors of MCL1. Values are IC50s (M).

| **compound name** | **IC50(M)** | **Library origin** |
| --- | --- | --- |
| aaah-MCL-2 | 8.19E-07 | large |
| aaau-MCL-4 | >5.00E-05 | large |
| aaau-MCL-9 | 2.21E-07 | large |
| aaap-MCL-11 | 4.58E-06 | large |
| aamt-MCL-13 | >5.00E-05 | large |
| aaah-MCL-16 | 1.35E-05 | large |
| aaah-MCL-17 | >5.00E-05 | large |
| aaae-MCL-18 | 2.82E-05 | large |
| aaah-MCL-19 | 4.06E-05 | large |
| aabp-MCL-25 | 3.49E-06 | large |
| aaqe-MCL-26 | >5.00E-05 | large |
| aaah-MCL-28 | >5.00E-05 | large |
| aabp-MCL-29 | 2.28E-05 | large |
| aaad-MCL-7 | 1.89E-05 | small |
| aaad-MCL-9 | 5.01E-05 | small |
| aaad-MCL-10 | 1.23E-05 | small |

**Supplementary Table 14.** SMILES strings of *N*-terminally acetylated *C*-terminally methyl amidated monomers used in the work.

| monomer name | SMILES strings | chemotype |
| --- | --- | --- |
| TIQ | CC(=O)N1[C@H](c(cccc2)c2CC1)C(=O)NC | a |
| ACHA14T | CC(=O)N[C@H]1CC[C@@H](CC1)CC(=O)NC | e |
| B2ALA | CC(=O)NC[C@H](C)C(=O)NC | b |
| OZR | CC(=O)N[C@@H](C)C1=N[C@H](CO1)C(=O)NC | r |
| NMLEU | CC(=O)N(C)[C@H](CC(C)C)C(=O)NC | a |
| PTABU | CC(=O)N(CC)CC(=O)NC | a |
| PPS | CC(=O)N1[C@H](C[C@H](c2ccccc2)C1)C(=O)NC | a |
| TZS | CC(=O)N[C@H](C)C1=N[C@H](CS1)C(=O)NC | r |
| TZR | CC(=O)N[C@@H](C)C1=N[C@H](CS1)C(=O)NC | r |
| PPR | CC(=O)N1[C@H](C[C@@H](c2ccccc2)C1)C(=O)NC | a |
| PRO | CC(=O)N1[C@H](CCC1)C(=O)NC | a |
| BAZE | CC(=O)N1CC(C1)C(=O)NC | b |
| B3PHG | CC(=O)N[C@H](c1ccccc1)CC(=O)NC | b |
| B3PHE | CC(=O)N[C@H](Cc1ccccc1)CC(=O)NC | a |
| INIP | CC(=O)N1CCC(CC1)C(=O)NC | g |
| PRR | CC(=O)N1C[C@H](CC1)C(=O)NC | a |
| PIP | CC(=O)N1[C@H](CCCC1)C(=O)NC | a |
| SAR | CC(=O)N(C)CC(=O)NC | a |
| AMC14C | CC(=O)NC[C@H]1CC[C@H](CC1)C(=O)NC | e |
| TZL | CC(=O)N[C@H](C)c1nc(cs1)C(=O)NC | t |
| GPN | CC(=O)NCC1(CCCCC1)CC(=O)NC | g |
| GAPC | CC(=O)N[C@@H]1C[C@@H](N(C(=O)C)C1)C(=O)NC | g |
| NAP2 | CC(=O)N[C@H](Cc1ccc(cccc2)c2c1)C(=O)NC | a |
| VAL | CC(=O)N[C@H](C(C)C)C(=O)NC | a |
| VIC | CC(=O)N[C@H](CCNC(=O)CC1)C(=O)N[C@@H]1C(=O)NC | r |
| ACHA14C | CC(=O)N[C@H]1CC[C@H](CC1)CC(=O)NC | e |
| THP | CC(=O)N1[C@H](CSC1)C(=O)NC | a |
| GAPT | CC(=O)N[C@H]1C[C@@H](N(C(=O)C)C1)C(=O)NC | g |
| AGLR | CC(=O)N[C@H](CC1)C(=O)N1[C@@H](C)C(=O)NC | r |
| AGLS | CC(=O)N[C@H](CC1)C(=O)N1[C@H](C)C(=O)NC | r |
| AGLY | CC(=O)NCC(=O)NC | a |
| D4ALA | CC(=O)N[C@H](C)CCCC(=O)NC | d |
| ACHC12T | CC(=O)N[C@H]1[C@@H](CCCC1)C(=O)NC | b |
| PHG | CC(=O)N[C@H](c1ccccc1)C(=O)NC | a |
| PHE | CC(=O)N[C@H](Cc1ccccc1)C(=O)NC | a |
| ACHC12C | CC(=O)N[C@H]1[C@H](CCCC1)C(=O)NC | b |
| GGLY | CC(=O)NCCCC(=O)NC | g |
| ACPenC | CC(=O)NC(CCC1)(C1)C(=O)NC | a |
| OZS | CC(=O)N[C@H](C)C1=N[C@H](CO1)C(=O)NC | r |
| PTIPA | CC(=O)N(C(C)C)CC(=O)NC | a |
| NIP | CC(=O)N1C[C@H](CCC1)C(=O)NC | g |
| ACBC13T | CC(=O)N[C@H]1C[C@@H](C1)C(=O)NC | g |
| NMABU | CC(=O)N(C)[C@H](CC)C(=O)NC | a |
| AMACBEN4 | CC(=O)NCc1ccc(cc1)CC(=O)NC | q |
| DGLY | CC(=O)NCCCCC(=O)NC | d |
| ACPC12C | CC(=O)N[C@H]1[C@H](C1)C(=O)NC | b |
| ACBC13C | CC(=O)N[C@H]1C[C@H](C1)C(=O)NC | g |
| ACBEN3 | CC(=O)Nc1cc(ccc1)CC(=O)NC | l |
| ACBEN2 | CC(=O)Nc1c(cccc1)CC(=O)NC | i |
| NMASN | CC(=O)N(C)[C@H](CC(=O)N)C(=O)NC | a |
| ACBEN4 | CC(=O)Nc1ccc(cc1)CC(=O)NC | o |
| ACPenC13T | CC(=O)N[C@H]1C[C@@H](CC1)C(=O)NC | g |
| HUCP | CC(=O)NCc1nc(ccc1)C(=O)NC | t |
| AMP | CC(=O)N1[C@](C)(CCC1)C(=O)NC | a |
| ACPenC13C | CC(=O)N[C@H]1C[C@H](CC1)C(=O)NC | g |
| TIC | CC(=O)N1[C@H](Cc(cccc2)c2C1)C(=O)NC | a |
| ACPhenC | CC(=O)NC(Cc(cccc2)c12)(C1)C(=O)NC | a |
| ACBC12T | CC(=O)N[C@H]1[C@@H](CC1)C(=O)NC | b |
| AMACBEN3 | CC(=O)NCc1cc(ccc1)CC(=O)NC | p |
| AMACBEN2 | CC(=O)NCc1c(cccc1)CC(=O)NC | m |
| B3ALA | CC(=O)N[C@H](C)CC(=O)NC | b |
| ACHC13C | CC(=O)N[C@H]1C[C@H](CCC1)C(=O)NC | g |
| NMPHG | CC(=O)N(C)[C@H](c1ccccc1)C(=O)NC | a |
| PTAMBA | CC(=O)N([C@@H](c1ccccc1)C)CC(=O)NC | a |
| AMBEN4 | CC(=O)NCc1ccc(cc1)C(=O)NC | n |
| ACBC12C | CC(=O)N[C@H]1[C@H](CC1)C(=O)NC | b |
| AMBEN3 | CC(=O)NCc1cc(ccc1)C(=O)NC | k |
| AMBEN2 | CC(=O)NCc1c(cccc1)C(=O)NC | h |
| ABOC | CC(=O)NC(CC1)(CCC1C2)[C@@H]2C(=O)NC | b |
| ACPC12T | CC(=O)N[C@H]1[C@@H](C1)C(=O)NC | b |
| ACHC14T | CC(=O)N[C@H]1CC[C@@H](CC1)C(=O)NC | d |
| OXO | CC(=O)N1[C@H](CCC1(=O))C(=O)NC | a |
| LARE | CC(=O)N[C@@H](C(=O)N)CCC(=O)NC | g |
| LARD | CC(=O)N[C@@H](C(=O)N)CC(=O)NC | b |
| TBA | CC(=O)N[C@H](CC(C)(C)C)C(=O)NC | a |
| G4ALA | CC(=O)N[C@H](C)CCC(=O)NC | g |
| NVL | CC(=O)N[C@H](CCC)C(=O)NC | a |
| ACHC13T | CC(=O)N[C@H]1C[C@@H](CCC1)C(=O)NC | g |
| TBG | CC(=O)N[C@H](C(C)(C)C)C(=O)NC | a |
| AMC14T | CC(=O)NC[C@H]1CC[C@@H](CC1)C(=O)NC | e |
| PTANI | CC(=O)N(c1ccccc1)CC(=O)NC | a |
| IDC | CC(=O)N1[C@H](Cc(cccc2)c12)C(=O)NC | a |
| B3LEU | CC(=O)N[C@H](CC(C)C)CC(=O)NC | b |
| SER | CC(=O)N[C@H](C[OH])C(=O)NC | a |
| BPIP | CC(=O)N1[C@H](CCCC1)CC(=O)NC | b |
| ASN | CC(=O)N[C@H](CC(=O)N)C(=O)NC | a |
| CLICK | CC(=O)NCc1cn(nn1)CC(=O)NC | v |
| NMPHE | CC(=O)N(C)[C@H](Cc1ccccc1)C(=O)NC | a |
| PYR3 | CC(=O)N[C@H](Cc1ccc(ccc3(ccc4))c(c3c4cc2)c12)C(=O)NC | a |
| CLA | CC(=O)N[C@H](C[Cl])C(=O)NC | a |
| ACHC | CC(=O)NC(CCCC1)(C1)C(=O)NC | a |
| EGLY | CC(=O)NCCCCCC(=O)NC | e |
| BEN3 | CC(=O)Nc1cc(ccc1)C(=O)NC | f |
| BEN2 | CC(=O)Nc1c(cccc1)C(=O)NC | c |
| BEN4 | CC(=O)Nc1ccc(cc1)C(=O)NC | j |
| ACPeneC13C | CC(=O)N[C@H]1C[C@H](C=C1)C(=O)NC | g |
| OZL | CC(=O)N[C@H](C)c1nc(co1)C(=O)NC | t |
| ACPenC12T | CC(=O)N[C@H]1[C@@H](CCC1)C(=O)NC | b |
| ALA | CC(=O)N[C@H](C)C(=O)NC | a |
| NMALA | CC(=O)N(C)[C@H](C)C(=O)NC | a |
| LEU | CC(=O)N[C@H](CC(C)C)C(=O)NC | a |
| ACPC | CC(=O)NC(C1)(C1)C(=O)NC | a |
| ACPenC12C | CC(=O)N[C@H]1[C@H](CCC1)C(=O)NC | b |
| SUGA | CC(=O)N[C@@H](C(=O)N)CSCC(=O)NC | s |
| AZE | CC(=O)N1[C@H](CC1)C(=O)NC | a |
| HUCQ | CC(=O)Nc1c2nc(ccc2ccc1)C(=O)NC | u |
| AIB | CC(=O)NC(C)(C)C(=O)NC | a |
| ANT9 | CC(=O)N[C@H](Cc1c(cccc2)c2cc(cccc2)c12)C(=O)NC | a |
| NAP1 | CC(=O)N[C@H](Cc1cccc(cccc2)c12)C(=O)NC | a |
| NHM | CC(=O)N1[C@H](Cc2c3ccccc3[nH]c2C1)C(=O)NC | a |
| B3VAL | CC(=O)N[C@H](C(C)C)CC(=O)NC | b |
| BGLY | CC(=O)NCCC(=O)NC | b |
| NMVAL | CC(=O)N(C)[C@H](C(C)C)C(=O)NC | a |
| ACBC | CC(=O)NC(CC1)(C1)C(=O)NC | a |
| FPA | CC(=O)N[C@H](Cc1c(F)c(F)c(F)c(F)c1(F))C(=O)NC | a |
| HPS | CC(=O)N1[C@H](C[C@@H](O)C1)C(=O)NC | a |
| HPR | CC(=O)N1[C@H](C[C@H](O)C1)C(=O)NC | a |
| DHA | CC(=O)NC(=C)C(=O)NC | a |
| ACHC14C | CC(=O)N[C@H]1CC[C@H](CC1)C(=O)NC | d |
| ABU | CC(=O)N[C@H](CC)C(=O)NC | a |
| FPS | CC(=O)N1[C@H](C[C@@H](F)C1)C(=O)NC | a |
| FPR | CC(=O)N1[C@H](C[C@H](F)C1)C(=O)NC | a |
| DHP | CC(=O)N1[C@H](C=CC1)C(=O)NC | a |
| BPRO | CC(=O)N1[C@H](CCC1)CC(=O)NC | b |
| NMSER | CC(=O)N(C)[C@H](C[OH])C(=O)NC | a |
| NMBEN4 | CC(=O)N(C)c1ccc(cc1)C(=O)NC | j |
| PTTBA | CC(=O)N(C(C)(C)C)CC(=O)NC | a |
| CLICKS | CC(=O)NCc1cn(nn1)[C@H](C)C(=O)NC | v |
| NMBEN2 | CC(=O)N(C)c1c(cccc1)C(=O)NC | c |
| NMBEN3 | CC(=O)N(C)c1cc(ccc1)C(=O)NC | f |

**Supplementary Table 15.** HPLC percent purity of each compound.

| compound name | HPLC % by area |
| --- | --- |
| aaad-MCL-7 | 90.8 |
| aaad-MCL-9 | 98.7 |
| aaad-MCL-10 | 95.15 |
| aaae-HDACi-1 | 77.4 |
| aaae-HDACi-2 | 97.81 |
| aaae-MCL-18 | 87.81 |
| aaag 1 | 89.6 |
| aaag 1 | 94.68 |
| aaah-MCL-2 | 98.84 |
| aaah-MCL-16 | 95.71 |
| aaah-MCL-17 | 96.67 |
| aaah-MCL-19 | 98.39 |
| aaah-MCL-28 | 98.96 |
| aaam 4 | 99.65 |
| aaam 5 | 98.45 |
| aaam 6 | 95.19 |
| aaam 8 | 98.04 |
| aaam 1 | 95.83 |
| aaam 2 | 97.41 |
| aaap 1 | 96.41 |
| aaap 1 | 97.53 |
| aaap 3 | 76 |
| aaap 4 | 99.4 |
| aaap-HDACi-3 | 95.43 |
| aaap-MCL-11 | 97.45 |
| aaaq 4 | 98.98 |
| aaau-MCL-4 | 95.99 |
| aaau-MCL-9 | 98.51 |
| aabb 1 | 96.09 |
| aabc 1 | 97.79 |
| aabc 2 | 99.02 |
| aabg 1 | 97.27 |
| aabi 2 | 98.97 |
| aabi 3 | 99.51 |
| aabi 4 | 96.05 |
| aabi 5 | 95.32 |
| aabi-MPRO-1 | 96.51 |
| aabi-MPRO-1g | 96.5 |
| aabi-MPRO-1g-linear | 99.33 |
| aabi-MPRO-1ga | 87.64 |
| aabi-MPRO-1gb | 93.88 |
| aabi-MPRO-1gc | 95.49 |
| aabi-MPRO-1gd | 85.87 |
| aabi-MPRO-1j-linear | 99.3 |
| aabi-MPRO-1ja | 89.12 |
| aabi-MPRO-1jb | 95.34 |
| aabi-MPRO-1jc | 87.01 |
| aabi-MPRO-1jd | 98.15 |
| aabi-MPRO-1je | 98.53 |
| aabi-MPRO-1k | 77.07 |
| aabi-MPRO-1k-linear | 98.99 |
| aabi-MPRO-1ka | 90.12 |
| aabi-MPRO-1kc | 97.35 |
| aabi-MPRO-1kd | 90.24 |
| aabi-MPRO-1ke | 97.21 |
| aabp-MCL-25 | 93.82 |
| aabp-MCL-29 | 95 |
| aabs 1 | 97.05 |
| aaeb-HDACi-5 | 98.1 |
| aagb_1 | 99.23 |
| aahb-HDACi-4 | 92.49 |
| aahb-HDACi-9 | 95.89 |
| aahb-HDACi-10 | 96.69 |
| aalm 1 | 99.36 |
| aamt-MCL-13 | 97.64 |
| aapb-HDACi-6 | 99.37 |
| aapb-HDACi-8 | 98.37 |
| aapb-HDACi-11 | 97.53 |
| aaqe-MCL-26 | 88.68 |
| aark-HDACi-7 | 97.27 |
| aas 1 | 97.69 |
| ahah 1 | 99.51 |
| akak 1 | 98.03 |
| asbc 3 | 87.837 |
| bbbb 3 | 95.04 |
| ahah 2 | 98.12 |

**Analytical HPLC and LCMS mass spectra of macrocycles**

*Analytical UPLC and LCMS mass spectrum of aas 1*

*Analytical UPLC and LCMS mass spectrum of akak 1*

*Analytical UPLC and LCMS mass spectrum of ahah 1*

*Analytical UPLC and LCMS mass spectrum of aabc 1*

*Analytical UPLC and LCMS mass spectrum of aaam 1*

*Analytical UPLC and LCMS mass spectrum of aaam 2*

*Analytical UPLC and LCMS mass spectrum of aaap 4*

*Analytical UPLC and LCMS mass spectrum of aaaq 1*

*Analytical UPLC and LCMS mass spectrum of aabc 2*

*Analytical UPLC and LCMS mass spectrum of aalm 1*

*Analytical HPLC and LCMS mass spectrum of aaam 4*

*Analytical HPLC and LCMS mass spectrum of aaam 5*

*Analytical HPLC and LCMS mass spectrum of aaam 6*

*Analytical HPLC and LCMS mass spectrum of aaam 8*

*Analytical HPLC and LCMS mass spectrum of aabs 1*

*Analytical HPLC and LCMS mass spectrum of aaap 1*

*Analytical HPLC and LCMS mass spectrum of aaap 2*

*Analytical HPLC and LCMS mass spectrum of aaap 3*

*Analytical HPLC and LCMS mass spectrum of asbc 3*

*Analytical UPLC and LCMS mass spectrum of aabb 1*

*Analytical UPLC and LCMS mass spectrum of aaag 1*

*Analytical UPLC and LCMS mass spectrum of aaag 4*

*Analytical HPLC and LCMS mass spectrum of aabg 1*

*Analytical HPLC and LCMS mass spectrum of aabi 2*

*Analytical HPLC and LCMS mass spectrum of aabi 3*

*Analytical HPLC and LCMS mass spectrum of aabi 4*

*Analytical HPLC and LCMS mass spectrum of aabi 5*

*Analytical UPLC and LCMS mass spectrum of bbbb 3*

*Analytical UPLC and LCMS mass spectrum of aagb 1*

*Analytical UPLC and LCMS mass spectrum of aaae-HDACi-1*

*Analytical UPLC and LCMS mass spectrum of aaae-HDACi-2*

*Analytical UPLC and LCMS mass spectrum of aaap-HDACi-3*

*Analytical UPLC and LCMS mass spectrum of aahb-HDACi-4*

*Analytical UPLC and LCMS mass spectrum of aaeb-HDACi-5*

*Analytical UPLC and LCMS mass spectrum of aapb-HDACi-6*

*Analytical UPLC and LCMS mass spectrum of aark-HDACi-7*

*Analytical UPLC and LCMS mass spectrum of aapb-HDACi-8*

*Analytical UPLC and LCMS mass spectrum of aahb-HDACi-9*

*Analytical UPLC and LCMS mass spectrum of aahb-HDACi-10*

*Analytical UPLC and LCMS mass spectrum of aapb-HDACi-11*

*Analytical UPLC and LCMS mass spectrum of ahah 2*

*Analytical UPLC and LCMS mass spectrum of aabi-MPRO-1k*

*Analytical UPLC and LCMS mass spectrum of aabi-MPRO-1g-linear*

*Analytical UPLC and LCMS mass spectrum of aabi-MPRO-1j-linear*

*Analytical UPLC and LCMS mass spectrum of aabi-MPRO-1k-linear*

*Analytical UPLC and LCMS mass spectrum of aabi-MPRO-1ja*

*Analytical UPLC and LCMS mass spectrum of aabi-MPRO-1ga*

*Analytical UPLC and LCMS mass spectrum of aabi-MPRO-1ka*

*Analytical UPLC and LCMS mass spectrum of aabi-MPRO-1jb*

*Analytical UPLC and LCMS mass spectrum of aabi-MPRO-1gb*

*Analytical UPLC and LCMS mass spectrum of aabi-MPRO-1gc*

*Analytical UPLC and LCMS mass spectrum of aabi-MPRO-1kc*

*Analytical UPLC and LCMS mass spectrum of aabi-MPRO-1kd*

*Analytical UPLC and LCMS mass spectrum of aabi-MPRO-1je*

*Analytical UPLC and LCMS mass spectrum of aabi-MPRO-1gd*

*Analytical UPLC and LCMS mass spectrum of aabi-MPRO-1jc*

*Analytical UPLC and LCMS mass spectrum of aabi-MPRO-1ke*

*Analytical UPLC and LCMS mass spectrum of aabi-MPRO-1jd*

*Analytical UPLC and LCMS mass spectrum of aabi-MPRO-1g*

*Analytical UPLC and LCMS mass spectrum of aabi-MPRO-1*

*Analytical UPLC and LCMS mass spectrum of aaah-MCL-2*

*Analytical UPLC and LCMS mass spectrum of aaau-MCL-4*

*Analytical UPLC and LCMS mass spectrum of aaau-MCL-9*

*Analytical UPLC and LCMS mass spectrum of aaap-MCL-11*

*Analytical UPLC and LCMS mass spectrum of aamt-MCL-13*

*Analytical UPLC and LCMS mass spectrum of aaah-MCL-16*

*Analytical UPLC and LCMS mass spectrum of aaah-MCL-17*

*Analytical UPLC and LCMS mass spectrum of aaae-MCL-18*

*Analytical UPLC and LCMS mass spectrum of aaah-MCL-19*

*Analytical UPLC and LCMS mass spectrum of aabp-MCL-25*

*Analytical UPLC and LCMS mass spectrum of aaqe-MCL-26*

*Analytical UPLC and LCMS mass spectrum of aaah-MCL-28*

*Analytical UPLC and LCMS mass spectrum of aabp-MCL-29*

*Analytical UPLC and LCMS mass spectrum of aaad-MCL-7*

*Analytical UPLC and LCMS mass spectrum of aaad-MCL-9*

*Analytical UPLC and LCMS mass spectrum of aaad-MCL-10*

Variable temperature ^1^H NMR studies of macrocycles

^1^H NMR studies of macrocycles

**References**

1.	V. Sarojini, A. J. Cameron, K. G. Varnava, W. A. Denny, G. Sanjayan, Cyclic Tetrapeptides from Nature and Design: A Review of Synthetic Methodologies, Structure, and Function. Chem. Rev. 119, 10318–10359 (2019).

2.	J. T. Mhlongo, E. Brasil, B. G. de la Torre, F. Albericio, Naturally Occurring Oxazole-Containing Peptides. Mar. Drugs. 18 (2020), doi:10.3390/md18040203.

3.	T. Degenkolb, W. Gams, H. Brückner, Natural cyclopeptaibiotics and related cyclic tetrapeptides: structural diversity and future prospects. Chem. Biodivers. 5, 693–706 (2008).

4.	M. J. Ferracane, A. C. Brice-Tutt, J. S. Coleman, G. G. Simpson, L. L. Wilson, S. O. Eans, H. M. Stacy, T. F. Murray, J. P. McLaughlin, J. V. Aldrich, Design, synthesis, and characterization of the macrocyclic tetrapeptide cyclo[Pro-Sar-Phe-d-Phe]: A mixed opioid receptor agonist-antagonist following oral administration. ACS Chem. Neurosci. 11, 1324–1336 (2020).

5.	E. M. Driggers, S. P. Hale, J. Lee, N. K. Terrett, The exploration of macrocycles for drug discovery — an underexploited structural class. Nat. Rev. Drug Discov. 7, 608–624 (2008).

6.	M. Muttenthaler, G. F. King, D. J. Adams, P. F. Alewood, Trends in peptide drug discovery. Nat. Rev. Drug Discov. 20, 309–325 (2021).

7.	D. Garcia Jimenez, V. Poongavanam, J. Kihlberg, Macrocycles in Drug Discovery─Learning from the Past for the Future. J. Med. Chem. 66, 5377–5396 (2023).

8.	Y. Goto, H. Suga, The RaPID Platform for the Discovery of Pseudo-Natural Macrocyclic Peptides. Acc. Chem. Res. 54, 3604–3617 (2021).

9.	K. Josephson, A. Ricardo, J. W. Szostak, mRNA display: from basic principles to macrocycle drug discovery. Drug Discov. Today. 19, 388–399 (2014).

10.	K. T. Mortensen, T. J. Osberger, T. A. King, H. F. Sore, D. R. Spring, Strategies for the Diversity-Oriented Synthesis of Macrocycles. Chem. Rev. 119, 10288–10317 (2019).

11.	G. Sangouard, A. Zorzi, Y. Wu, E. Ehret, M. Schüttel, S. Kale, C. Díaz-Perlas, J. Vesin, J. Bortoli Chapalay, G. Turcatti, C. Heinis, Picomole‐scale synthesis and screening of macrocyclic compound libraries by acoustic liquid transfer. Angew. Chem. Weinheim Bergstr. Ger. 133, 21870–21875 (2021).

12.	S. Habeshian, M. L. Merz, G. Sangouard, G. K. Mothukuri, M. Schüttel, Z. Bognár, C. Díaz-Perlas, J. Vesin, J. Bortoli Chapalay, G. Turcatti, L. Cendron, A. Angelini, C. Heinis, Synthesis and direct assay of large macrocycle diversities by combinatorial late-stage modification at picomole scale. Nat. Commun. 13, 3823 (2022).

13.	A. Roy, E. Koesema, T. Kodadek, High-Throughput Quality Control Assay for the Solid-Phase Synthesis of DNA-Encoded Libraries of Macrocycles*. Angew. Chem. Int. Ed Engl. 60, 11983–11990 (2021).

14.	S. A. Rettie, K. V. Campbell, A. K. Bera, A. Kang, S. Kozlov, J. De La Cruz, V. Adebomi, G. Zhou, F. DiMaio, S. Ovchinnikov, G. Bhardwaj, Cyclic peptide structure prediction and design using AlphaFold. bioRxiv (2023), doi:10.1101/2023.02.25.529956.

15.	J. L. Watson, D. Juergens, N. R. Bennett, B. L. Trippe, J. Yim, H. E. Eisenach, W. Ahern, A. J. Borst, R. J. Ragotte, L. F. Milles, B. I. M. Wicky, N. Hanikel, S. J. Pellock, A. Courbet, W. Sheffler, J. Wang, P. Venkatesh, I. Sappington, S. V. Torres, A. Lauko, V. De Bortoli, E. Mathieu, S. Ovchinnikov, R. Barzilay, T. S. Jaakkola, F. DiMaio, M. Baek, D. Baker, De novo design of protein structure and function with RFdiffusion. Nature. 620, 1089–1100 (2023).

16.	P. Hosseinzadeh, G. Bhardwaj, V. K. Mulligan, M. D. Shortridge, T. W. Craven, F. Pardo-Avila, S. A. Rettie, D. E. Kim, D.-A. Silva, Y. M. Ibrahim, I. K. Webb, J. R. Cort, J. N. Adkins, G. Varani, D. Baker, Comprehensive computational design of ordered peptide macrocycles. Science. 358, 1461–1466 (2017).

17.	E. A. Coutsias, K. W. Lexa, M. J. Wester, S. N. Pollock, M. P. Jacobson, Exhaustive Conformational Sampling of Complex Fused Ring Macrocycles Using Inverse Kinematics. J. Chem. Theory Comput. 12, 4674–4687 (2016).

18.	R. Zubatyuk, J. S. Smith, J. Leszczynski, O. Isayev, Accurate and transferable multitask prediction of chemical properties with an atoms-in-molecules neural network. Sci Adv. 5, eaav6490 (2019).

19.	E. Caldeweyher, S. Ehlert, A. Hansen, H. Neugebauer, S. Spicher, C. Bannwarth, S. Grimme, A generally applicable atomic-charge dependent London dispersion correction. J. Chem. Phys. 150, 154122 (2019).

20.	W. H. B. Sauer, M. K. Schwarz, Molecular shape diversity of combinatorial libraries: a prerequisite for broad bioactivity. J. Chem. Inf. Comput. Sci. 43, 987–1003 (2003).

21.	G. Bhardwaj, V. K. Mulligan, C. D. Bahl, J. M. Gilmore, P. J. Harvey, O. Cheneval, G. W. Buchko, S. V. S. R. K. Pulavarti, Q. Kaas, A. Eletsky, P.-S. Huang, W. A. Johnsen, P. Greisen Jr, G. J. Rocklin, Y. Song, T. W. Linsky, A. Watkins, S. A. Rettie, X. Xu, L. P. Carter, R. Bonneau, J. M. Olson, E. Coutsias, C. E. Correnti, T. Szyperski, D. J. Craik, D. Baker, Accurate de novo design of hyperstable constrained peptides. Nature. 538, 329–335 (2016).

22.	P. K. Madala, J. D. A. Tyndall, T. Nall, D. P. Fairlie, Update 1 of: Proteases universally recognize beta strands in their active sites. Chem. Rev. 110, PR1–31 (2010).

23.	C. J. Miller, B. E. Turk, Homing in: Mechanisms of Substrate Targeting by Protein Kinases. Trends Biochem. Sci. 43, 380–394 (2018).

24.	J. Gavenonis, B. A. Sheneman, T. R. Siegert, M. R. Eshelman, J. A. Kritzer, Comprehensive analysis of loops at protein-protein interfaces for macrocycle design. Nat. Chem. Biol. 10, 716–722 (2014).

25.	M. Guharoy, P. Chakrabarti, Secondary structure based analysis and classification of biological interfaces: identification of binding motifs in protein-protein interactions. Bioinformatics. 23, 1909–1918 (2007).

26.	B. L. Sibanda, T. L. Blundell, J. M. Thornton, Conformation of beta-hairpins in protein structures. A systematic classification with applications to modelling by homology, electron density fitting and protein engineering. J. Mol. Biol. 206, 759–777 (1989).

27.	P. N. Lewis, F. A. Momany, H. A. Scheraga, Chain reversals in proteins. Biochim. Biophys. Acta. 303, 211–229 (1973).

28.	C. A. Lipinski, F. Lombardo, B. W. Dominy, P. J. Feeney, Experimental and computational approaches to estimate solubility and permeability in drug discovery and development settings. Adv. Drug Deliv. Rev. 64, 4–17 (2012).

29.	G. Bhardwaj, J. O’Connor, S. Rettie, Y.-H. Huang, T. A. Ramelot, V. K. Mulligan, G. G. Alpkilic, J. Palmer, A. K. Bera, M. J. Bick, M. Di Piazza, X. Li, P. Hosseinzadeh, T. W. Craven, R. Tejero, A. Lauko, R. Choi, C. Glynn, L. Dong, R. Griffin, W. C. van Voorhis, J. Rodriguez, L. Stewart, G. T. Montelione, D. Craik, D. Baker, Accurate de novo design of membrane-traversing macrocycles. Cell. 185, 3520–3532.e26 (2022).

30.	G. J. Saunders, A. K. Yudin, Property-Driven Development of Passively Permeable Macrocyclic Scaffolds Using Heterocycles. Angew. Chem. Int. Ed Engl. 61, e202206866 (2022).

31.	A. A. Rzepiela, L. A. Viarengo-Baker, V. Tatarskii, R. Kombarov, A. Whitty, Conformational Effects on the Passive Membrane Permeability of Synthetic Macrocycles. J. Med. Chem. 65, 10300–10317 (2022).

32.	P. Hosseinzadeh, P. R. Watson, T. W. Craven, X. Li, S. Rettie, F. Pardo-Avila, A. K. Bera, V. K. Mulligan, P. Lu, A. S. Ford, B. D. Weitzner, L. J. Stewart, A. P. Moyer, M. Di Piazza, J. G. Whalen, P. Greisen Jr, D. W. Christianson, D. Baker, Anchor extension: a structure-guided approach to design cyclic peptides targeting enzyme active sites. Nat. Commun. 12, 3384 (2021).

33.	T. Muramatsu, C. Takemoto, Y.-T. Kim, H. Wang, W. Nishii, T. Terada, M. Shirouzu, S. Yokoyama, SARS-CoV 3CL protease cleaves its C-terminal autoprocessing site by novel subsite cooperativity. Proc. Natl. Acad. Sci. U. S. A. 113, 12997–13002 (2016).

34.	H. Park, G. Zhou, M. Baek, D. Baker, F. DiMaio, Force Field Optimization Guided by Small Molecule Crystal Lattice Data Enables Consistent Sub-Angstrom Protein–Ligand Docking. J. Chem. Theory Comput. 17, 2000–2010 (2021).

35.	J. W. Johannes, S. Bates, C. Beigie, M. A. Belmonte, J. Breen, S. Cao, P. A. Centrella, M. A. Clark, J. W. Cuozzo, C. E. Dumelin, A. D. Ferguson, S. Habeshian, D. Hargreaves, C. Joubran, S. Kazmirski, A. D. Keefe, M. L. Lamb, H. Lan, Y. Li, H. Ma, S. Mlynarski, M. J. Packer, P. B. Rawlins, D. W. Robbins, H. Shen, E. A. Sigel, H. H. Soutter, N. Su, D. M. Troast, H. Wang, K. F. Wickson, C. Wu, Y. Zhang, Q. Zhao, X. Zheng, A. W. Hird, Structure Based Design of Non-Natural Peptidic Macrocyclic Mcl-1 Inhibitors. ACS Med. Chem. Lett. 8, 239–244 (2017).

36.	J. Lyu, J. J. Irwin, B. K. Shoichet, Modeling the expansion of virtual screening libraries. Nat. Chem. Biol. 19, 712–718 (2023).

37.	D. B. Diaz, S. D. Appavoo, A. F. Bogdanchikova, Y. Lebedev, T. J. McTiernan, G. Dos Passos Gomes, A. K. Yudin, Illuminating the dark conformational space of macrocycles using dominant rotors. Nat. Chem. 13, 218–225 (2021).

38.	E. Koesema, A. Roy, N. G. Paciaroni, C. Coito, M. Tokmina-Roszyk, T. Kodadek, Synthesis and screening of a DNA‐encoded library of non‐peptidic macrocycles. Angew. Chem. Weinheim Bergstr. Ger. 134 (2022), doi:10.1002/ange.202116999.

39.	K. T. Butler, F. J. Luque, X. Barril, Toward accurate relative energy predictions of the bioactive conformation of drugs. J. Comput. Chem. 30, 601–610 (2009).

40.	A. C. Brueckner, Q. Deng, A. E. Cleves, C. A. Lesburg, J. C. Alvarez, M. Y. Reibarkh, E. C. Sherer, A. N. Jain, Conformational Strain of Macrocyclic Peptides in Ligand-Receptor Complexes Based on Advanced Refinement of Bound-State Conformers. J. Med. Chem. 64, 3282–3298 (2021).

41.	J. W. Liebeschuetz, The Good, the Bad, and the Twisted Revisited: An Analysis of Ligand Geometry in Highly Resolved Protein-Ligand X-ray Structures. J. Med. Chem. 64, 7533–7543 (2021).

42.	A. N. Jain, A. C. Brueckner, A. E. Cleves, M. Reibarkh, E. C. Sherer, A Distributional Model of Bound Ligand Conformational Strain: From Small Molecules up to Large Peptidic Macrocycles. J. Med. Chem. 66, 1955–1971 (2023).

43.	V. K. Mulligan, S. Workman, T. Sun, S. Rettie, X. Li, L. J. Worrall, T. W. Craven, D. T. King, P. Hosseinzadeh, A. M. Watkins, P. D. Renfrew, S. Guffy, J. W. Labonte, R. Moretti, R. Bonneau, N. C. J. Strynadka, D. Baker, Computationally designed peptide macrocycle inhibitors of New Delhi metallo-β-lactamase 1. Proc. Natl. Acad. Sci. U. S. A. 118 (2021), doi:10.1073/pnas.2012800118.

44.	Patrick Salveson, Adam Moyer, Meerit Said, Gizem Gocke, Xinting Li, Alex Kang, Hannah Nguyen, Asim Bera, Paul Levine, Gaurav Bhardwaj, David Baker, Data for Expansive discovery of chemically diverse structured macrocyclic oligoamides. Zenodo (2023), , doi:10.5281/zenodo.10109705.

45.	C. Bannwarth, S. Ehlert, S. Grimme, GFN2-xTB-an accurate and broadly parametrized self-consistent tight-binding quantum chemical method with multipole electrostatics and density-dependent dispersion contributions. J. Chem. Theory Comput. 15, 1652–1671 (2019).

46.	W. Kabsch, XDS. Acta Crystallogr. D Biol. Crystallogr. 66, 125–132 (2010).

47.	M. D. Winn, C. C. Ballard, K. D. Cowtan, E. J. Dodson, P. Emsley, P. R. Evans, R. M. Keegan, E. B. Krissinel, A. G. W. Leslie, A. McCoy, S. J. McNicholas, G. N. Murshudov, N. S. Pannu, E. A. Potterton, H. R. Powell, R. J. Read, A. Vagin, K. S. Wilson, Overview of the CCP4 suite and current developments. Acta Crystallogr. D Biol. Crystallogr. 67, 235–242 (2011).

48.	G. Sheldrick, SHELXT: Integrating space group determination and structure solution. Acta Crystallogr. A Found. Adv. 70, C1437–C1437 (2014).

49.	G. M. Sheldrick, Crystal structure refinement with SHELXL. Acta Crystallogr. B. 71, 3–8 (2015).

50.	P. Emsley, K. Cowtan, Coot: model-building tools for molecular graphics. Acta Crystallogr. D Biol. Crystallogr. 60, 2126–2132 (2004).

51.	C. B. Hübschle, G. M. Sheldrick, B. Dittrich, ShelXle: a Qt graphical user interface for SHELXL. J. Appl. Crystallogr. 44, 1281–1284 (2011).

52.	A. M. Tsimberidou, P. A. Beer, C. A. Cartwright, C. Haymaker, H. H. Vo, S. Kiany, A. R. L. Cecil, J. Dow, K. Haque, F. A. Silva, L. Coe, H. Berryman, E. A. Bone, G. M. Nogueras-Gonzalez, D. Vining, H. McElwaine-Johnn, I. I. Wistuba, Preclinical Development and First-in-Human Study of KA2507, a Selective and Potent Inhibitor of Histone Deacetylase 6, for Patients with Refractory Solid Tumors. Clin. Cancer Res. 27, 3584–3594 (2021).

File S1.

Zip Archive of relevant source code required for execution of the methods described in the manuscript and SI
